# MEvA-X: a hybrid multiobjective evolutionary tool using an XGBoost classifier for biomarkers discovery on biomedical datasets

**DOI:** 10.1093/bioinformatics/btad384

**Published:** 2023-06-16

**Authors:** Konstantinos Panagiotopoulos, Aigli Korfiati, Konstantinos Theofilatos, Peter Hurwitz, Marco Agostino Deriu, Seferina Mavroudi

**Affiliations:** PolitoBIOMed Lab, Department of Mechanical and Aerospace Engineering, Politecnico di Torino, Corso Duca degli Abruzzi 24, Turin, 10129, Italy; Intelligent Systems Biology (InSyBio) PC, Patras Science Park building Platani, Patras, 26504, Greece; Intelligent Systems Biology (InSyBio) PC, Patras Science Park building Platani, Patras, 26504, Greece; School of Cardiovascular and Metabolic Medicine & Sciences, King's College, London, SE5 9NU, United Kingdom; Clarity Science LLC, 750 Boston Neck Road Suite 11, Narragansett, Rhode Island, 02882, United States; PolitoBIOMed Lab, Department of Mechanical and Aerospace Engineering, Politecnico di Torino, Corso Duca degli Abruzzi 24, Turin, 10129, Italy; Intelligent Systems Biology (InSyBio) PC, Patras Science Park building Platani, Patras, 26504, Greece; Department of Nursing, School of Health Rehabilitation Sciences, University of Patras, University campus, Rio, Achaia, 26504, Greece

## Abstract

**Motivation:**

Biomarker discovery is one of the most frequent pursuits in bioinformatics and is crucial for precision medicine, disease prognosis, and drug discovery. A common challenge of biomarker discovery applications is the low ratio of samples over features for the selection of a reliable not-redundant subset of features, but despite the development of efficient tree-based classification methods, such as the extreme gradient boosting (XGBoost), this limitation is still relevant. Moreover, existing approaches for optimizing XGBoost do not deal effectively with the class imbalance nature of the biomarker discovery problems, and the presence of multiple conflicting objectives, since they focus on the training of a single-objective model. In the current work, we introduce MEvA-X, a novel hybrid ensemble for feature selection (FS) and classification, combining a niche-based multiobjective evolutionary algorithm (EA) with the XGBoost classifier. MEvA-X deploys a multiobjective EA to optimize the hyperparameters of the classifier and perform FS, identifying a set of Pareto-optimal solutions and optimizing multiple objectives, including classification and model simplicity metrics.

**Results:**

The performance of the MEvA-X tool was benchmarked using one omics dataset coming from a microarray gene expression experiment, and one clinical questionnaire-based dataset combined with demographic information. MEvA-X tool outperformed the state-of-the-art methods in the balanced categorization of classes, creating multiple low-complexity models and identifying important nonredundant biomarkers. The best-performing run of MEvA-X for the prediction of weight loss using gene expression data yields a small set of blood circulatory markers which are sufficient for this precision nutrition application but need further validation.

**Availability and implementation:**

https://github.com/PanKonstantinos/MEvA-X.

## 1 Introduction

Due to the exponential increase of computational power in the last decades, life sciences and biology increasingly rely on informatics to tackle the complexity of the systems they examine. In addition, in the time of -omics, where the feature space is vast and the number of samples is usually small, finding reliable nonredundant biomarkers is one of the most frequent quests for scientists (Aronson and Ferner 2017). Biomarkers are useful indicators for the early detection of various pathologies such as neurodegenerative diseases, and different types of cancer and can help in the surveillance of the progression of these pathologies with low-cost and minimally invasive techniques (Chahine *et al.* 2014, Ganepola *et al.* 2014, Leuzy *et al.* 2022). Even with previous technologies such as microarrays, the number of genes detected—ranging from 2000 up to more than 20 000—is many times greater than the number of samples (Mukhopadhyay and Mandal 2014, Boucheham *et al.* 2015). Bioinformatics emerged from these needs, aiming to bridge the gap between those fields by introducing new computational tools tailored to the demands of biomedical applications (Manisekhar *et al.* 2020).

In the last decades, many algorithms have tried to deal with the complexity of real-life problems which possess multiple and conflicting objectives. Hybrid wrapper and ensemble classification techniques have been developed by combining evolutionary algorithms (EAs) and machine learning methods such as k-nearest neighbors, artificial neural networks (Salari *et al.* 2014), and others (Rapakoulia *et al.* 2014, Kleftogiannis *et al.* 2015). Heuristic and metaheuristic techniques have been introduced that search for acceptable solutions in a wide feature space without providing any mathematical proof for the optimality of the revealed solutions (Swiercz *et al.* 2014, Boucheham *et al.* 2015). Inspired by the theory of evolution in nature, EAs are among the most used methods in these cases with multiple variations being proposed over the years (Corthésy *et al.* 2018, Lambora *et al.* 2019, Chen *et al.* 2020, Katoch *et al.* 2021). Multiobjective EA can be combined with Pareto techniques that apply selective pressure to yield a set of equally good solutions, instead of trying to find the global best, since the nature of these problems does not allow for a trivial or easy way to converge to one solution only (Abraham and Jain 2005). Moreover, it is important to allow the exploration of a wide set of the dominant solutions of the Pareto optimal set to avoid premature convergence in nonoptimal solutions. One of the successful techniques that have been proposed to deal with these requirements is niching, where solutions of the same Pareto set get penalized according to their similarity (Fonseca and Fleming 1993). Recently, a novel gradient-boosting technique called XGBoost (Chen and Guestrin 2016) has been proposed, and since then it has gained a lot of attention in computer science challenges hosted by Kaggle competitions.

It has been demonstrated that the combination of an XGBoost model with a genetic algorithm (GA) can substantially enhance its performance by allowing for the search for the optimal hyperparameters of the classifier (Chen *et al.* 2020). Despite XGBoost classifiers performing reasonably well with their default parameters on various datasets, optimizing their parameters in an unbiased way is important for achieving higher performance. XGBoost possesses many hyperparameters and their effect on the performance of the trained models is important. Deng *et al.* (2022) proposed a similar method where the XGBoost algorithm was used as an ensemble-based feature selection (FS) method to get a subset of the initial number of genes, which later they used as input for the GA they use for training multiobjective models. Even though this approach showed promising results, as a greedy algorithm, XGBoost may neglect genes that might have some statistical meaning.

In their work, Corthésy *et al.* (2018) implemented a similar solution to the proposed method, but with a different classifier.

XGBoost has already been applied alone in many bioinformatics and biomedical applications (Li *et al.* 2022, Ma *et al.* 2022) while the use of metaheuristics for optimizing their hyperparameters has also been successfully performed in many other applications (Desdhanty and Rustam 2021, Ghatasheh *et al.* 2022, Syed *et al.* 2022). However, most of these approaches do not deal with multiple objectives and are not able to effectively handle the data missingness, the class imbalance, and the high dimensionality of the data which are used for the discovery of predictive biosignatures. Multiobjective EAs have already been applied for this purpose but without using XGBoost as a classifier. For instance, a support vector classifier was used as an estimator (Corthésy *et al.* 2018), neglecting at that time the potential discrimination power of boosting algorithms. In a more recent application, a novel framework, named AUTODC (Bai *et al.* 2022), has been introduced and tested for disease classification using other boosting and tree-based classification and optimizing them with a novel heuristic method based on a two-layer Multi-Armed Bandit framework. This method outperformed existing tools for biomarker discovery by effectively selecting features and hyperparameters for the classification datasets. However, it neither included XGBoost in the classification methods nor provided a solution for multiple objectives optimization, class imbalance, or multiple solutions for the same problem.

In the present work, we introduce MEvA-X, a multiobjective hybrid ensemble EA framework for optimizing the hyperparameters of an XGBoost classifier for biomarkers discovery applications. The boosting algorithm has been selected as a binary discriminator, and its hyperparameters get optimized over the generations of the EA. In the presented method, we use a niched Pareto frontier scheme, which helps to conserve the diversity of the solutions by distributing the population over several different peaks (niches) and avoiding premature convergence of the algorithm (Horn *et al.* 1994). This allows for a general-purpose biomarker discovery tool that works with numerical features not only for omics but also for clinical features. MEvA-X was benchmarked against other established state-of-the-art methods, such as the XGBoost algorithm alone, Random Forests, and XGBoost combined with other standard FS techniques to validate its performance.

Two publicly available datasets were used to test the proposed method for evaluation purposes. The first dataset is from a study that investigated the relationship between weight loss through lifestyle interventions and gene expression profiles in peripheral blood (Blackburn *et al.* 2015, Ellsworth *et al.* 2015). This study collected data over the span of 1 year from people with high cardiac risk. Our interest was to use the baseline measurements at the beginning of the study and create models to predict if a person will lose weight with the examined intervention, as well as to find biomarkers that can help us decide if this intervention will be suitable for other subject based on their gene expression profile. Toward this goal, MEvA-X retrieved 24 solutions from the dominant Pareto frontier and exceeded 75% receiver operating characteristic (AUC) with the best performing one including only nine selected genes, and significantly improved the classification of the minority class compared with the state-of-the-art methods considering that this is an imbalanced dataset with a very low samples-to-features ratio.

The second dataset is linked to the retrospective analysis of a population study on 631 chronic pain patients who were prescribed opioid painkillers, and who were treated with topical analgesics formulations. The information contained in this dataset comes from the answers of the subjects in the Brief Pain Inventory (BPI) (Cleeland and Ryan 1994) at the beginning (baseline) and at the follow-up period, and it is encoded in numerical scaled values. The study aimed to document changes in 3 months in four categories: BPI pain severity, pain interference relating to Quality of Life components, other medication usage, and the qualitative health complaints of these patients (Gudin *et al.* 2017).

In this dataset, MEvA-X achieved high improvement for all four labels, since the imbalance is even greater which favors the proposed method compared with other techniques. There was an increase of 16%–32% in the weighted geometric mean (wGM) in absolute numbers and an 8%–10% increase in balanced accuracy, while 8–19 features were used for the four labels.

Several configurations and combinations of the parameters for the EA were tested to evaluate the whole pipeline and to assess the robustness of the method against the baseline and the models with a selected subset of features (see Supplementary File). In all these cases, we found that MEvA-X classification outperforms the state-of-the-art methods while providing a nonredundant set of features as biomarkers.

## 2 Materials and methods

### 2.1 Datasets

The first dataset was obtained by merging two different data sources downloaded from the Gene Expression Omnibus (GEO) databank. In greater detail, the accession number GSE66175 (Blackburn *et al.* 2015) contains 26 patients, plus another 63 patients who are collected from an older dataset (GSE46097) (Ellsworth *et al.* 2014), and 71 control subjects. The above-mentioned 89 patient subjects in total, were people having either a coronary artery disease (CAD) event or being at high risk of developing a CAD, following a therapeutic strategy characterized by a combination of an active lifestyle and diet modification targeting a relevant change in their weight in 3 and 12 months. The active lifestyle changes consisted of 3 h/week of aerobic exercise and 1 h of daily stress management, while the diet modification refers to a low-fat vegetarian diet known as Ornish. Blood samples were drawn at the beginning of the study (baseline) and later during the re-examination periods. The gene expression levels in peripheral whole blood samples were measured for these patients using the Affymetrix Human Genome U133A 2.0 Array platform (Blackburn *et al.* 2015).

The two patient cohorts contained in GSE66175 have been unified by a batch effect correction method (Zhang *et al.* 2020), and the duplicated gene names were grouped, taking their average values. Therefore, the merged dataset consists of 89 patients. In this dataset, weight loss information transformed into a binary label: participants with a weight loss higher than a selected threshold equal to 10% were considered to belong in the positive class (Responders), whereas participants with lower weight loss or even increased weight were considered to belong in the negative class (Non_responders). Based on the above-defined threshold, the label distribution of the dataset is 35 “Non_responders” and 54 “Responders,” with 13 239 unique gene and transcript names.

The second dataset included data from the Optimizing Patient Experience and Response to Topical Analgesics (OPERA) study. The OPERA dataset consists of 631 patients with chronic pain in the intervened group, answering the BPI-validated questionnaire along with some supplementary questions before and after a follow-up period (Gudin *et al.* 2017) for a total of 50 survey questions as features. Therefore, this is a dataset made of 631 patients each with 50 features.

The target of the researchers releasing the OPERA dataset was to investigate the effect of replacing opioid therapies with topical analgesics on patients with chronic pain and record the changes in multiple aspects of their life such as pain and medicine reduction, interference with everyday activities, and reduction of complaints. Consequently, four different labels in this dataset refer to the changes the scientist measured during the study (see Supplementary Table S1), and so all four of them were considered as different datasets and used to validate the performance of the proposed method.

### 2.2 Methods

**Figure 1.**
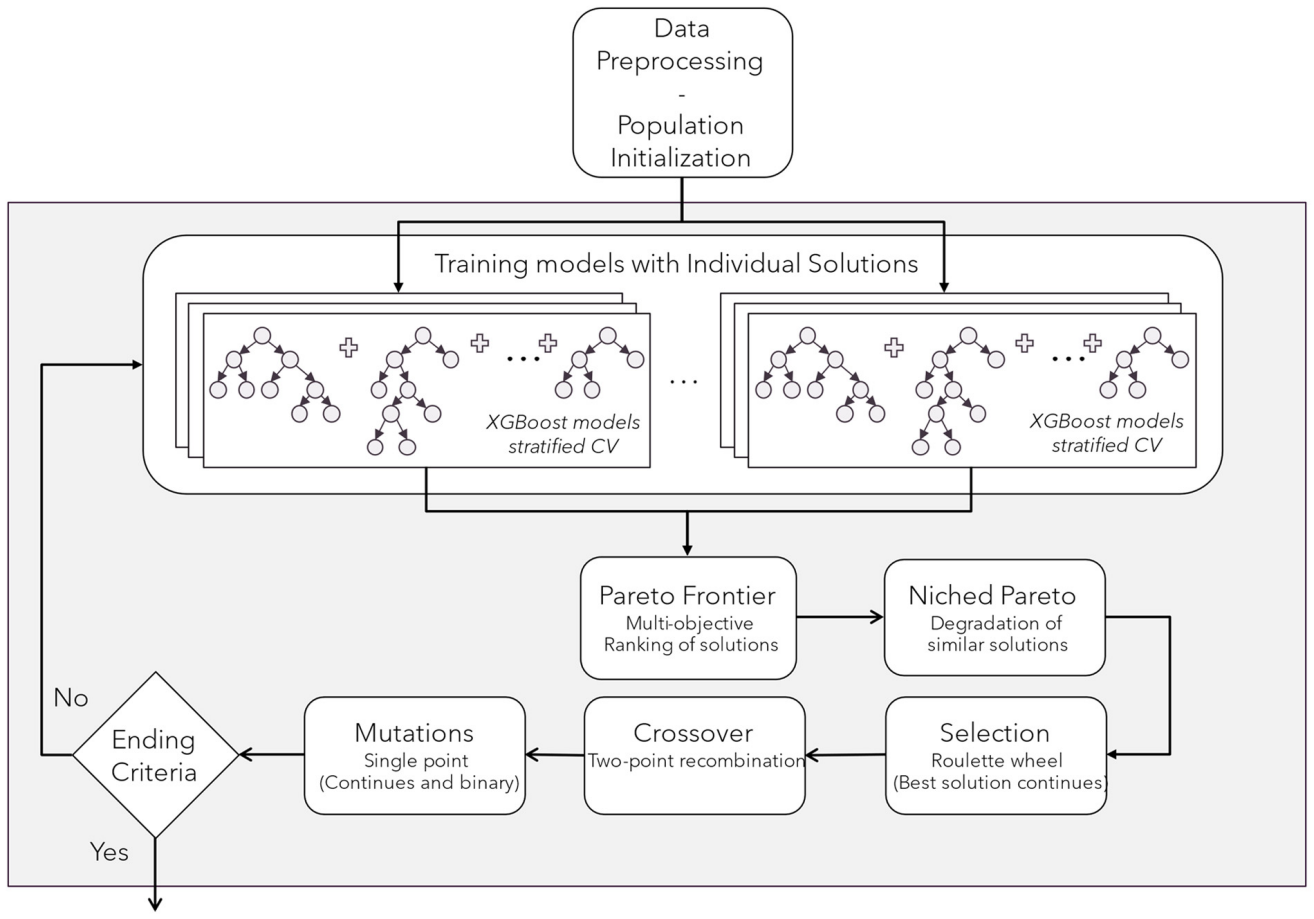
Flow chart of MEvA-X. The evolutionary process begins with data preprocessing and population initialization. The collection of individual solutions that encode the information in the form of chromosomes is used to train independent ensemble models using the XGBoost classifier in a 10-fold cross-validation framework. The solutions are ranked in frontiers through the Pareto Frontier method and similar solutions belonging in the same niche are degraded. The evolutionary operations (selection, crossover, and mutation) apply on the solutions and if the end criteria are not met the procedure starts over.

A hybrid ensemble algorithm for FS and classification, based on a multiobjective EA (Fig. 1) has been developed and introduced. MEvA-X optimizes the hyperparameters of an XGBoost Classifier while selecting nonredundant features (potential biomarkers) to reduce the dimensionality of the given problem. The presented method follows four individual main steps which are: data preprocessing, training of individual models, evaluation of the trained models, and population update based on the evolutionary processes.

Specifically, MEvA-X, except the main framework of the EA with the known operators (selection, crossover, mutation), implements a niched Pareto frontier ranking scheme which makes it appropriate for searching big feature spaces without converging to one local minimum (Horn *et al.* 1994). The niches are essentially different peaks (local maxima) in the objective function and this method keeps a relative balance between the distribution of the solution in these valleys to preserve pluralism and promote the searching of the feature space. After this ranking, the selection of solutions indicates which of the individuals in the population will pass their genes to the next generations through their offspring by recombining their chromosomes in pairs in the crossover operation. The mutation operator is also allowed in the offspring introducing further stochasticity in the process to allow better exploration of the search space.

The preprocessing steps of the pipeline are described in detail in the Supplementary Methods and include the transformation of nominal values to numeric, imputation of missing values, normalization of the features, and merging of duplicated features.

### 2.3 The MeVA-X evolutionary framework

#### 2.3.1 Feature selection

As an additional preprocessing step, features are selected using four different univariate and multivariate FS methods, namely, SelectKBest, Wilcoxon Rank Sums, Joint mutual information (JMI), and Minimum Redundancy Maximum Relevance (mRMR) (Rosner *et al.* 2003, Vergara and Estévez 2014). This way some of the solutions of the population can randomly select features from a lower-dimensional space which is based on statistical methods and can help in creating some niches (local minima) to improve the search for good feasible solutions. In the chromosome of each solution, there are specific “genes” that drive the individual to select features based on some FS methods and other “genes” responsible for the parameters of these methods.

#### 2.3.2 Initialization of population

The population of the first generation is generated in a pseudorandom manner by selecting values for the parameter genes following a uniform distribution for the range of the minimum and maximum allowed values for every parameter. The selection and activation of feature genes is also a random process, but the initial total number of active genes in any individual chromosome is constrained to less than 30, which are then filtered according to the FS genes.

#### 2.3.3 Stratified cross-validation data splitting

A stratified 10-fold cross-validation scheme with a different random state in every generation is used in *MEvA-X*, splitting the data into training and validation sets to provide concrete results of the trained models’ performance dealing effectively with the imbalanced nature of most of the clinical and biological datasets. In this way, the results are less prone to biases caused by single splits and the ratio of the positive and negative classes is preserved while keeping the same random state across the given generation allowing for comparable results between the solutions.

#### 2.3.4 Classification and hyperparameter tuning

In the presented method, XGBoost classifiers are used to discriminate the samples of the dataset according to the labels. The instructions to build each classifier are encoded in every individual solution in the last seven parameter genes (see Supplementary Table S2). The information contained in the chromosomes included seven hyperparameters of the classifier, namely the learning rate, the maximum depth of each tree, two pruning parameters, two generalization parameters, and a balancing parameter.

In every iteration of the cross-validation, the XGBoost classifier denotes the performance of both training and validation sets on the area under the AUC curve. In the cases where the validation AUC does not improve for more than 50 iterations, the training is terminated to avoid overfitting and the algorithm returns the ensemble up to the last best validation iteration.

#### 2.3.5 Network creation and bioinformatics analysis

The analysis of the data was made with the programming languages Python version 3.9 and R version 4.1.2. For the Ornish diet dataset, a coexpression network constructed with the help of InSyBio BioNets (Theofilatos *et al.* 2016) tool using the Spearman correlation (Artusi *et al.* 2002) and node PageRank centrality (Coppola *et al.* 2019) metrics for the selection of the most significant nodes. The visualization and further analysis of the network were done with Cytoscape (Shannon *et al.* 2003) version 3.9.1 and the tissue specificity analysis was conducted with the GTExPortal (Lonsdale *et al.* 2013). Additionally, the Spearman correlation and the principal component analysis (PCA) (Abdi and Williams 2010) were also calculated for the selected features of the MEvA-X models for both datasets. The results of these analyses can be seen in Fig. 3.

#### 2.3.6 Fitness functions

Based on the evolutionary operators, the solutions of the population “compete” to improve their fitness and survive in every iteration/generation of the algorithm, and the competition is held based on the objectives the EA ultimately tries to optimize. In MEvA-X, the multiple objectives mostly refer to the metrics used to evaluate the performance of the trained XGBoost classifier models, which are coordinated by the chromosomes of the individual solutions in the population. The coordination is done by the parameter genes and the feature genes of the solutions, which are the blueprints for the construction of the discrimination models.

The proposed method is designed to maintain a balance between the high discrimination performance and the low complexity of the models. Thus, the evolutionary pressure drives the population towards more sparse chromosomes with as few active feature genes as possible, while maintaining high classification performance. Feature_model_complexity and Split_model_complexity are used to measure the complexity of the trained models. The value of these metrics is lower for complex models and higher for simpler ones. Particularly, the more active gene features a solution has, the lower the feature model complexity metric will be. Similarly, a model with fewer splits and a simpler structure will have a greater split complexity score than a model with more trees in the ensemble and many splits.

On the other hand, metrics that evaluate the discrimination of the models based on the prediction and the probabilities of the predictions of the classes are used as well. More specifically, accuracy, wGM, F1 score, F2 score, precision, recall, balanced accuracy, and the AUC were considered as evaluation metrics.

Finally, the overall score and the weighted overall score are also calculated to give an easy comparison between the solutions. The overall score considers each of the previously referred metrics as of equal importance, while the weighted overall score is a user’s choice array of weights mapped to the metrics based on the importance of each one of them. This way, the user of MEvA-X has an additional degree of freedom to drive the population toward solutions with higher scores on the preferred objectives.

#### 2.3.7 Niched Pareto frontier

The trained models are ranked based on their multiple evaluation metrics through a Pareto Frontier operation (Abraham and Jain 2005). A niched Pareto frontier ranking approach has been selected like the proposal of Erickson *et al.* (2001) to avoid the premature convergence of the algorithm and for a more exhaustive search of the feature and parameter space. This way similar solutions that belong to the same Pareto front get penalized even if they reach a very high score. In this manner, the pluralism of solutions is guaranteed during the passage of generations and local maxima are avoided.

In MEvA-X, there is a hybrid approach of binary and continuous encoding on the chromosomes, and so the calculation of the distance between two solutions belonging to the same Pareto frontier is a two-step process.

For measuring the difference between two individual solutions belonging to the same Pareto front, we introduced a dual distance metric. One part of the metric refers to the continuous values of the parameters to be optimized and the second to the binary selection of features of the dataset (see Supplementary Methods). According to the closeness of the solutions, a degradation function is applied for similar solutions to reduce the possibility of the algorithm accumulating all solutions in a local minimum.

The applied evolutionary operators, termination criteria, and additional details on the evolutionary framework of MEvA-X are provided in Supplementary Methods.

### 2.4 Training final ensemble classification models

When at least one of the stop criteria is fulfilled, the EA stops operating. Then, the final solutions are ranked once more with the Pareto frontier method. The models that end up in the first Pareto frontier are the dominant ones and the solution with the highest overall score is the best compromise. Except for the overall best, it is possible to take advantage of the contradictive objectives and so the dominant solutions are combined to create an ensemble of classifiers with two majority voting methods. In the first method, the final prediction of the ensemble is the one that has the highest number of votes from the individual models in the ensemble, which is also known as “hard” majority voting. The second method implied is the so-called “soft” majority voting which considers the probability the XGBoost classifier gives to the prediction. In both cases, a filtering of the solutions participating in the voting is taking place, because some of the models end up in the first Pareto frontier simply because they are very low in complexity, but they might also have no discrimination value to add to the ensemble, and so solutions that classified all instances in the same class in the cross-validation, are excluded from the majority voting.

## 3 Results

**Figure 2.**
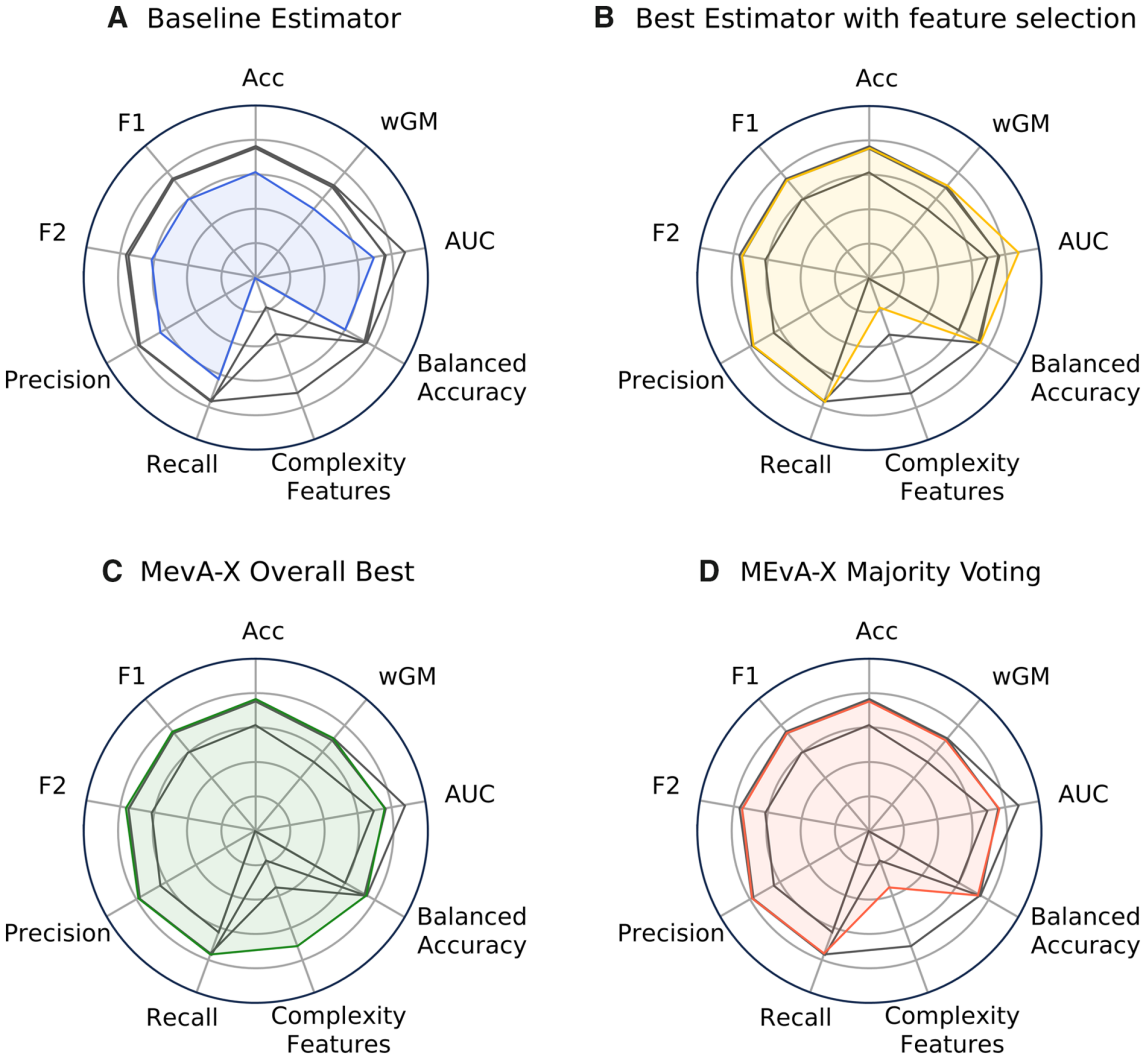
Comparative radar plots between the average performance of models for the Ornish diet dataset. (A) Simple XGBoost estimator, (B) best model with FS applied (JMI with *k* = 5), (C) MEvA-X models with the highest overall metric in the population, and (D) majority voting of the solutions in the first Pareto frontier. The metrics and their standard deviations calculated from the 10-fold cross-validation analysis are provided in the Supplementary File (Supplementary Table S5). Each panel colors the metrics of each presented method and shows in gray the rest.

The performance and robustness of MEvA-X have been compared with the existing state-of-the-art XGBoost classifier by several experiments that were conducted with both datasets (Ornish diet and OPERA) using the stratified 10-fold cross-validation framework. In these experiments, MEvA-X’s performance was benchmarked against single XGBoost models (baseline) and XGBoost models trained on a subset of the features with the FS techniques of Wilcoxon ranked sum, SelectKBest, JMI, and mRMR. For the comparison with the state-of-the-art methods, a radar plot was used to visualize the improvement of the models created by MEvA-X in terms of simplicity and balance in the discrimination of classes (Fig. 2).

**Figure 3.**
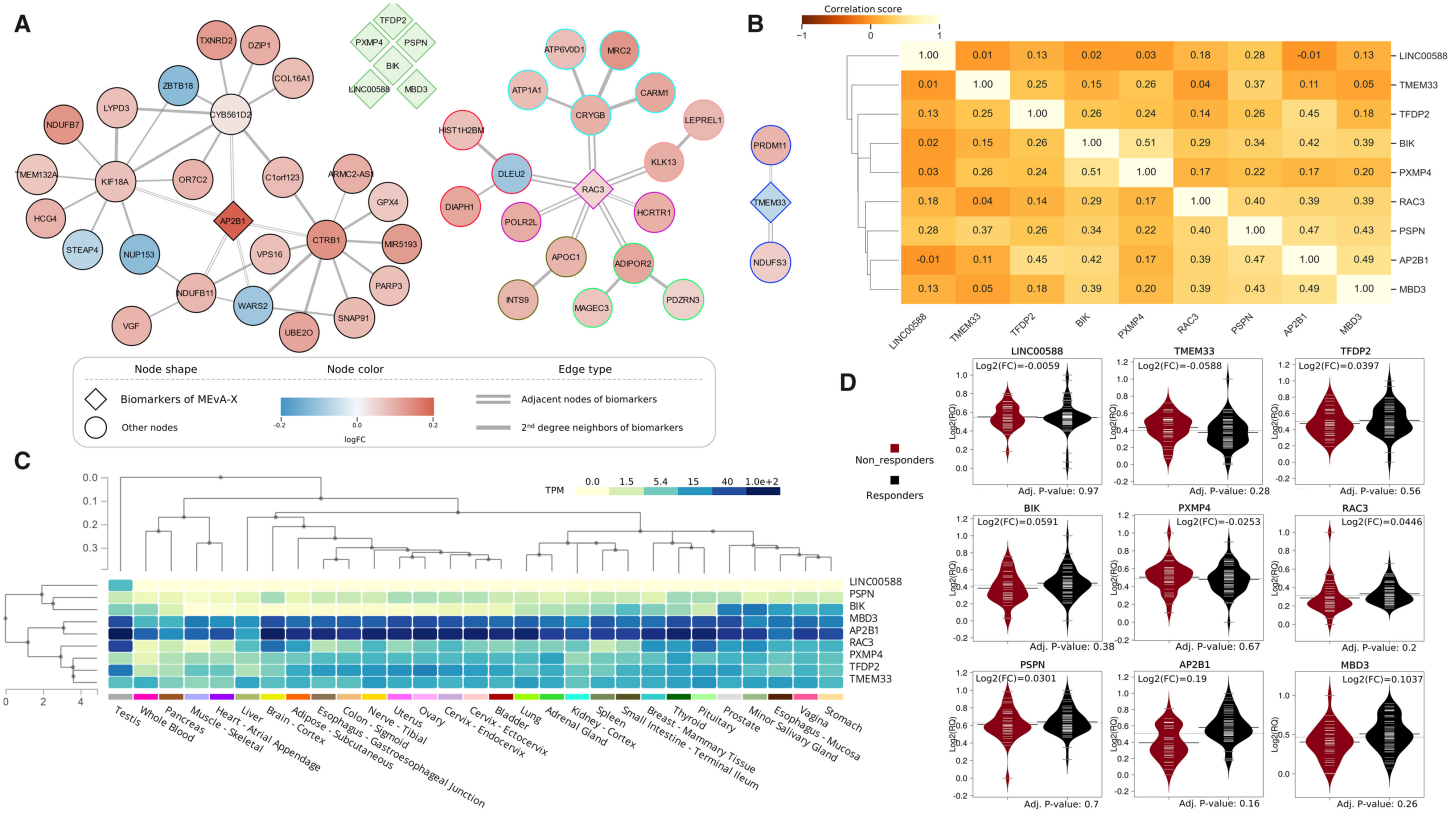
Bioinformatics analysis of the MEvA-X precision nutrition biosignature. (A) The coexpression network of the selected features and their immediate and two-steps-away neighbors, with the border color of the nodes representing the clusters in the network. (B) Correlation matrix of the selected features using Spearman’s correlation. (C) Expression of selected genes on different tissues and organs (https://gtexportal.org). (D) Violin plots of the distribution of the selected features based on the label (responders/nonresponders).

A coexpression network of the selected features and their neighborhoods was reconstructed, the distribution of the features was visualized, a correlation heatmap of the selected features was created, and a tissue specificity analysis was conducted for the revealed Ornish diet weight-loss prediction biosignature to interpret and explore the patterns and associations between the gene expression biomarkers (Fig. 3).

Additionally, enrichment analysis has been conducted to reveal any underlying pathways but for the given set of biomarkers, no term was found enriched. Furthermore, a PCA and the contribution of the features in the loadings of PCA were calculated and presented in Supplementary Figs S1–S5.

To benchmark the MEvA-X against another established method, we used Random Forests combined with a grid search for parameter optimization. Comparative results, as shown in Supplementary Tables S5 and S6, demonstrated that MEvA-X was able to substantially increase classification metrics, with a balanced accuracy increase from 61% to 76%.

**Figure 4.**
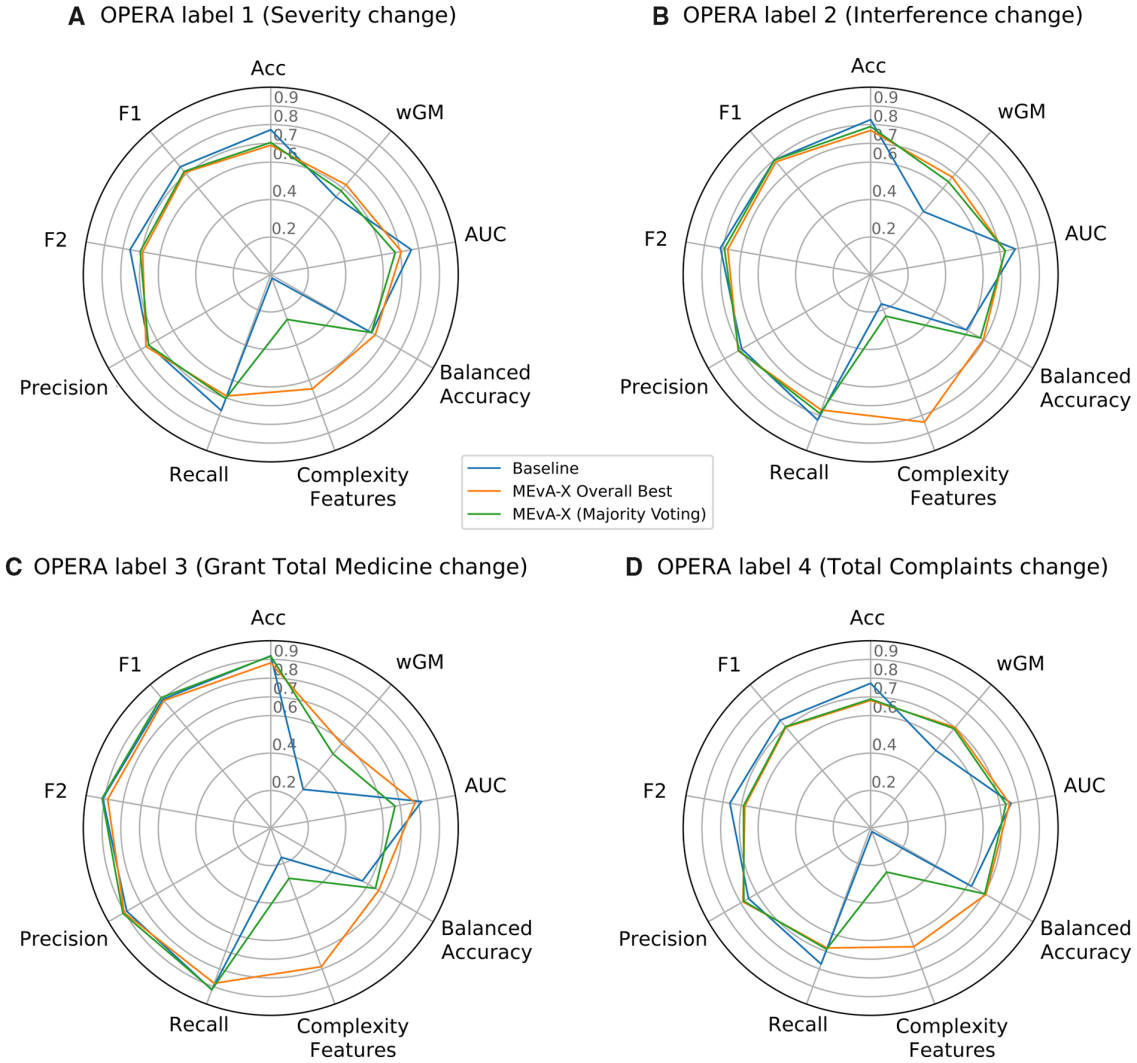
Comparative radar plots between the baseline and the MEvA-X solutions for the four endpoints of the OPERA dataset. MEvA-X outperforms the baseline models, both in simplicity (number of features used) and in the discrimination power of the minority class. (A) Endpoint related with the chainge in pain severity over the follow-up period. (B) Endpoint related to the difference in the interference of pain in everyday tasks over the follow-up period. (C) Endpoint showing the change of the total prescribed medicine to patients over the follow-up period. (D) Endpoint depicting the change of complaints of the patients over the course of the follow-up period. The metrics and their standard deviations calculated from the 10-fold cross-validation analysis are provided in the Supplementary File (Supplementary Table S7).

The proposed algorithm was tested also on the four chronic pain-related endpoints of the second (OPERA) dataset using data from a questionnaire survey. As before, radar plots were used to depict the evaluation metrics of the different frameworks against the MEvA-X tool (Fig. 4).

**Figure 5.**
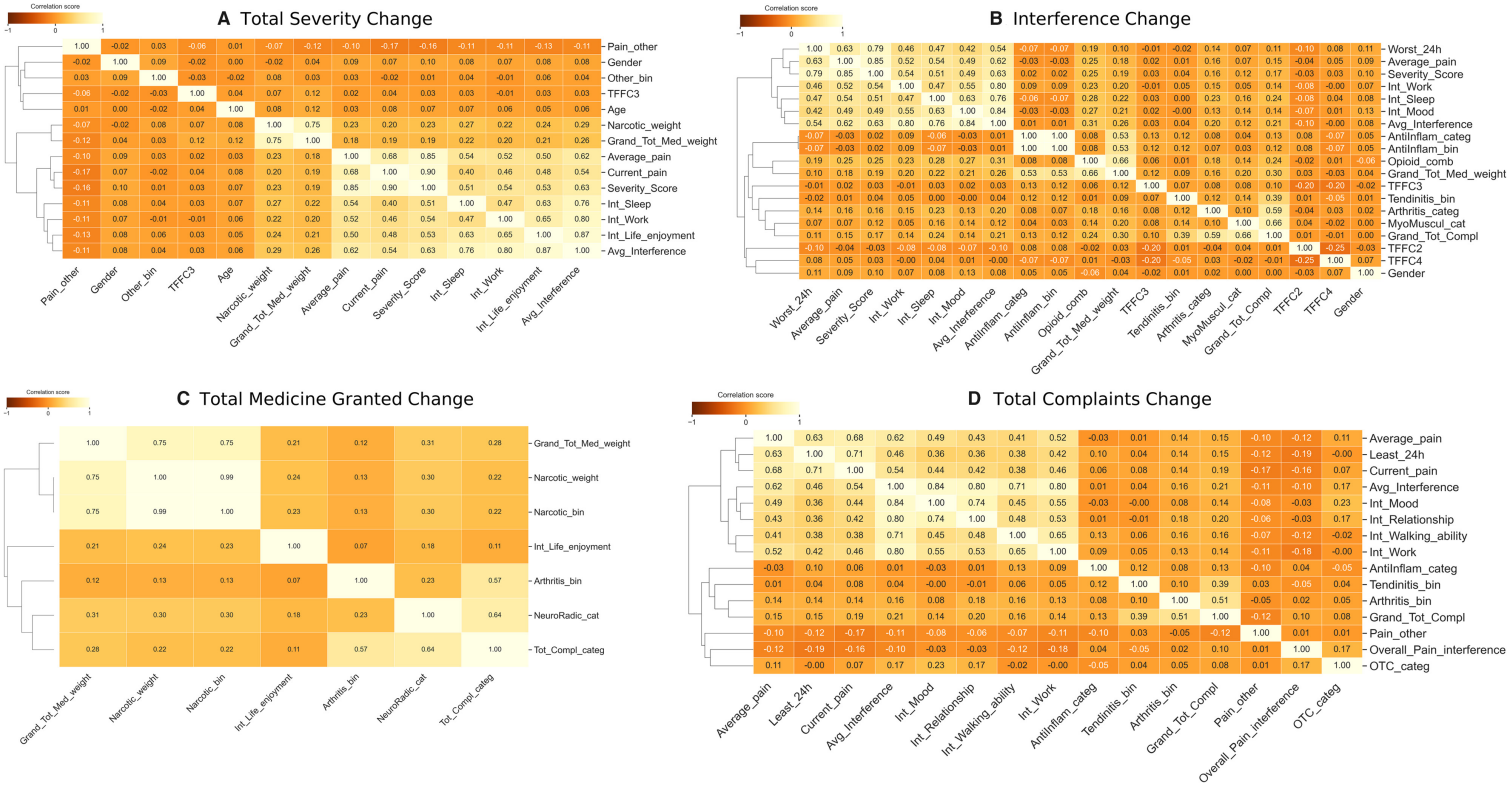
Correlation of the selected features by MEvA-X, for the OPERA dataset for the four labels. (A) Label 1 “Total Severity Change.” (B) Label 2 “Interference Change.” (C) Label 3 “Grant Total Medicine Change.” (D) Label 4 “Total Complaints Change.” Spearman’s correlation method was used and plots depict the Spearman’s Rho coefficient.

Α Spearman’s correlation feature association analysis was conducted for the features selected for all four endpoints of the dataset as shown in the heatmaps in Fig. 5. For the individual endpoints, MEvA-X selected a different set of features. For the *Severity Change* endpoint, a subset of 14 features was selected as important, while for the second endpoint (*Change in* Interference), 19 features were selected (Supplementary Table S9).

Regarding the third endpoint in the dataset (Change in Medicines), the algorithm ended up in a subset of eight features, and finally, in the last label (Change in Complaints) 15 features remained in the subset (Supplementary Table S9).

Further analysis of the principal components made for the OPERA datasets along with a feature importance ranking of the principal component loadings are provided in Supplementary Figs S6–S25.

MEvA-X increased the performance in the classification of the minority class with features that have low intercorrelation, leading to fewer nonredundant markers and relatively simple models.

## 4 Discussion

From the obtained results, it was shown that the MEvA-X algorithm is beneficial in two major aspects of biomedical classification problems. Optimizing the hyperparameters and features of the XGBoost classifier using the multiobjective optimization framework of MEvA-X resulted in the improvement of classification metrics, that are appropriate for imbalanced datasets (e.g. wGM) compared with XGBoost, XGboost coupled with FS methods, and grid searched optimized Random Forests. In both datasets used in the present work, the classification metrics significantly improved without a substantial corresponding decrease in the rest of the metrics. Moreover, MEvA-X increased substantially the simplicity of the final models and especially the model with the highest overall score yield by MEvA-X. The decrease in the complexity of the model is up to 60% in absolute numbers, meaning that the algorithm can identify features that improve the classification of the data without keeping redundant and low-informative ones. Especially for -omics datasets, such as the Ornish diet dataset, except for the very high features–samples ratio problem, there is high redundancy due to the coexpression of genes, making the identification of nonredundant biomarkers a challenge that our algorithm proved able to overcome.

Regarding the performance of MEvA-X on the correct classification of the minority class, it is superior to all benchmarked methods (see Supplementary Tables S3 and S4). Without the use of the EA, most of the instances are classified as the majority class resulting in very low wGM and balanced accuracy scores. Our algorithm can correct this misclassification in these difficult and imbalanced problems.

It is worth mentioning that the use of standard FS techniques alone was not proven to be efficient for both datasets. In the OPERA dataset, JMI and mRMR created very simple models of limited discrimination ability, while SelectKBest and Wilcoxon’s rank sum had poor performance in both datasets. XGBoost with no FS performed much better than the FS techniques for the OPERA dataset. Rapakoulia *et al.* (2014) showed that EAs optimized classification models are beneficial for problems with missing values when majority voting is used. A similar approach was adopted in MEvA-X, which enables the identification of multiple similarly performing prediction models. Majority voting was applied but the performance of this metaclassifier did not outperform the prediction performance of the best-performing model for each dataset, but no missing values were present in our datasets. Nevertheless, the training of multiple models with similar classification performance is extremely important for biomedical applications since different measurements are conducted in different patient cohorts with high missing values rate.

In the precision diet dataset, further bioinformatics analysis was performed on the selected biosignature set to interpret the results (Fig. 3). Based on the correlation analysis of the biosignatures, it appears that there is little correlation between them, indicating the effective selection of nonredundant features by the tool. This was confirmed when attempting to perform pathway and functional enrichment analysis with no term being significantly enriched in the revealed biosignature. Additionally, the coexpression network of the selected features with their first- and second-degree neighbors was reconstructed. Three of the selected features of MEvA-X (AP2B1, RAC3, and TMEM33) were identified as hubs according to a PageRank centrality-based analysis for their subnetworks, suggesting that these features could potentially be markers to indicate if this specific diet would be beneficial for a patient. Adapter-related protein complex 2 subunit beta 1 (AP2B1) encodes one of the large chain components of the assembly protein complex 2 whose functionality is to protein transport via transport vesicles in different membrane traffic pathways. Rac Family Small GTPase 3 (RAC3) encodes a protein that according to Gene Ontology is involved in GTP binding and calcium-dependent protein binding. Transmembrane Protein 33 (TMEM33) encodes a protein that is involved in the structural constituent of the nuclear pore and was found to maintain intracellular calcium homeostasis (Arhatte *et al.* 2019). Furthermore, from the tissue-specificity bioinformatics analysis, it is observed that these genes are not specific in metabolism-related tissues and organs. The non-coding LINC00588 and the BIK gene are gender-specific since they are mostly expressed in the testis and prostate, but the dataset is balanced between male and female subjects that eliminate the sex bias, so their changes are most likely explained from differential expression between responders and not responders within the male group. Even though most of the selected genes have a notable expression in the pancreas, thyroid, liver, and stomach, no previous association has been made between these markers and weight loss except one study identifying AP2B1 as a potential marker for eosinophilic gastroenteritis (Zhang *et al.* 2022), linking it thus indirectly to weight loss. These genes are associated with completely different molecular functions and pathways while their independent predictive potential is small with univariate statistical analysis showing marginal significance or no significant changes between responders and nonresponders (Fig. 3D). This suggests that the MEvA-x method was able to generate a highly accurate biosignature combining weak independent features, without being limited by the inherent assumptions of parametric tests for differential expression and removing redundancy from the selected features.

MEvA-X has the potential to become part of the Artificial Intelligence (AI) tools arsenal existing for solving difficult medical and biological problems. Advancements in AI have already allowed algorithms to be used in translational research applications having prospects in many diseases such as cancer, diabetes, and others (Bohr and Memarzadeh 2020, Rompianesi *et al.* 2022). Another very promising field for biomarker discovery is neurodegenerative diseases with substantial progress being made lately in conditions such as Alzheimer’s disease (Hansson 2021). This tool can help in the identification of potential biomarkers and can become part of a pipeline for the exploration of neuro diseases.

## Supplementary Material

btad384_Supplementary_Data

## Data Availability

The Ornish diet dataset was obtained from National Center for Biotechnology Information (NCBI) Gene Expression Omnibus (GEO) and is accessible through GEO Series accession number GSE66175 (https://www.ncbi.nlm.nih.gov/geo/query/acc.cgi?acc=GSE66175). The OPERA dataset was provided by Clarity Science LLC and is available upon request to the corresponding author with permission of Clarity Science LLC.
