# Supplementary material for: MEvA-X: a hybrid multiobjective evolutionary tool using an XGBoost classifier for biomarkers discovery on biomedical datasets
Source: Bioinformatics. 2023 Jun 16;39(7):btad384. doi: 10.1093/bioinformatics/btad384 (PMC10354005; doi:10.1093/bioinformatics/btad384)
Supplement: btad384_Supplementary_Data [file btad384_supplementary_data.docx]

Supporting file

MEvA-X: A Hybrid Multi-Objective Evolutionary Tool Using an XGBoost Classifier for Biomarkers Discovery on Biomedical Datasets

Konstantinos Panagiotopoulos^1^, Aigli Korfiati^2^, Konstantinos Theofilatos^2,3^, Peter Hurwitz^4^, Marco A. Deriu^1^ and Seferina Mavroudi^2,5^

^1^PoliTo BioMed Lab, Department of Mechanical and Aerospace Engineering, Politecnico di Torino, Turin, Italy

^2^Inteligent Systems Biology (InSyBio) PC, Patras Science Park building Platani, Patras, Greece

^3^School of Cardiovascular and Metabolic Medicine & Sciences, Kings College, London, United Kingdom

^4^Clarity Science LLC, Narragansett, Rhode Island USA

^5^Department of Nursing, School of Rehabilitation Sciences, University of Patras, Patras, Greece.

# Supporting Information

[Supporting Information 2](#_Toc134813452)

[Supplementary Methods 3](#_Toc134813453)

[**Preprocessing** 3](#_Toc134813454)

[**Initialization of population** 3](#_Toc134813455)

[**Calculation of distance between solutions** 3](#_Toc134813456)

[Evolutionary Operators 4](#_Toc134813457)

[Termination criteria 5](#_Toc134813458)

[Datasets 5](#_Toc134813459)

[**Ornish diet dataset** 5](#_Toc134813460)

[**OPERA dataset** 5](#_Toc134813461)

[Python Libraries and versions 5](#_Toc134813462)

[Bioinformatics analysis 5](#_Toc134813463)

[Random Forest optimized with Randomized Grid Search 5](#_Toc134813464)

[Metrics 6](#_Toc134813465)

[Algorithm 7](#_Toc134813466)

[Supplementary Figures 8](#_Toc134813467)

[Supplementary Tables 20](#_Toc134813468)

[References 24](#_Toc134813469)

# Supplementary Methods

## **Preprocessing**

The input data and labels for MEvA-X are transformed into numerical values, in case their original form is nominal or categorical, with the method of Label Encoding^1^. The dataset is searched for missing and abnormal values which then are imputed with a K-nearest neighbors [KNN] scheme^2^. Duplicated feature names are merged by getting their mean to have unique features in the dataset. Even though the normalization of the data is not necessary for the tree-based XGBoost classifier, for keeping a consistent pre-processing process with the other methods, the data are normalized feature-wise within the range [0,1] by the MinMaxScaler method^1,3^. The final product of the pre-processing method is a two-dimensional data frame object where the rows correspond to the features and the columns to the samples of the dataset, while the labels are kept in a different one-dimensional array corresponding to each example and have only numerical values.

## **Initialization of population**

The population of the first generation is created in a pseudo-random manner by selecting values for the parameter-genes following a uniform distribution for the range of the minimum and maximum allowed values for every parameter. The selection and activation of feature genes is also a random process, but the initial number of active genes in any individual chromosome is constrained to be less than 30 genes, and the selected feature-genes are then filtered according to the feature selection genes.

## **Calculation of distance between solutions**

**Distance of parameter-genes calculation:**

The distance of parameter genes between two solutions belonging to the same Pareto:

| $D_{pg}(a,b)=\sqrt{\frac{\sum_{i=1}^{\#param} \left( \frac{gene_{a,i}-gene_{b,i}}{\left( gene_{i} \right) -min(gene_{i})} \right)^{2}}{\#param}}$ | (1) |
| --- | --- |

**Distance of feature genes calculation:**

Logical XOR for the rounded gene values divided by the total number of unique active genes:

| $D_{fg}\left( a,b \right)=\frac{\sum_{j=1}^{\#Features} g}{gen{es}_{a}\cup gen{es}_{b}}$  where $g= \left\{ \begin{aligned} 1, if gene_{a,j} \neq gene_{b,j} \\ 0, if gene_{a,j}=gene_{b,j} \end{aligned} \right.$ | (2) |
| --- | --- |
|  |  |

**Pairwise Total distance:**

To calculate the total distance of two solutions that belong in the same Pareto frontier, the parameter distance ($D_{pg}$) and the feature distance ($D_{fg}$) are used to calculate their average.

| $D_{tot}\left( a,b \right)=\frac{D_{pg}+D_{fg}}{2}$ | (3) |
| --- | --- |

**Degradation of evaluation metrics: (niche (peak))**

In the proposed method, a Niched Pareto method that penalizes similar solutions is acting on the individuals that belong in the same Pareto frontier, is deployed to keep a balance between good solutions and pluralism.

| $\sigma_{share}=\frac{0.5}{\sqrt[10]{features}}$  ${m_{a}}_{shared}=\left\{ \begin{aligned} \sum_{b \in Pareto} \left( 1-\left( \frac{D_{tot}\left( a,b \right)}{\sigma_{share}} \right)^{2} \right), if D_{tot}\leq\sigma_{share} \\ 0, otherwise \end{aligned} \right.$ | (4) |
| --- | --- |
|  | (5) |
|  |  |

where $\sigma_{share}$ is the radius of the niche (neighborhood) around each solution.

Finally, the degradation of the solutions is calculated as:

| $f_{a_{shared}}=\frac{f_{pareto max}}{{m_{a}}_{shared}}$ | (6) |
| --- | --- |

where $f_{pareto max}$ is the highest values of the whole Pareto frontier the solution a belongs to, and ${m_{a}}_{shared}$ is the previously calculated shared factor based on the adjacency of solution a with the other solutions in the same Pareto frontier.

# Evolutionary Operators

Every EA has some basic operations that are applied to differentiate the initial population of solutions and allows these algorithms to converge to better solutions. Different approaches and variations have been proposed through the years, but the main operators are conserved as the building blocks of the algorithm, including Selection, Crossover and Mutation operators ^4,5^.

In our approach, the solution with the highest weighted overall score is considered the best solution of the generation and passes with its original chromosome unchanged to the next generation. Later, all solutions including the one with the highest overall score get a probability of being selected for pairing proportional to their weighted overall score over the total weighted overall score for all individual solutions.

Despite the numerous alternatives that have been proposed through the years, in MEvA-X the two-point cross-over was chosen as the recombination of genes for the two offspring chromosomes. The two-point approach translates into the exchange of a specific region of the parental genomes between two randomly defined points (P1, P2) with a fixed length (L). The crossover is an operation that is not necessarily happening in every mating of the parental genomes, and the probability of this operation is predefined at 90%; while the rest of the times (1 out of 10) there is no crossover whatsoever, and the parental chromosomes pass to the next generation unchanged.

For the mutation of the offspring, a probability of 5% is selected to allow for the exploration of the feature space without introducing dramatic changes in the population. Single point mutations -similar to SNPs- are used by MEvA-X in the feature genes, toggling the state of these genes from active to inactive and vice versa. The number of point mutations is determined randomly and is not allowed to be higher than six genes to change the new chromosome without bringing huge changes in it. For the parameter-genes on the other hand, since they are coded in continuous values, the mutations on these genes follow a Gaussian distribution around the mean of every parameter.

# Termination criteria

There are two conditions that whenever either one of them is met, the evolutionary algorithm terminates. The evolutionary algorithm in MEvA-X is terminated under two conditions and if either one of them is fulfilled. The first termination criterion is if the maximum number of generations selected by the user have reached, while the number of generations that have passed compared to the maximum generations number the user has selected. If the algorithm reaches the number of generations given by the user, it exits the evolutionary process and stores the final solutions. The second ending criterion is the convergence of the solutions to a single niche. This allows the algorithm to stop the evolutionary process if the population has reached very similar solutions and the evolutionary operations will not allow for further exploration of the feature and parameter space.

# Datasets

For the benchmarking of the MEvA-X tool, the Ornish diet and the OPERA datasets were used, respectively.

## **Ornish diet dataset**

The first dataset as it is presented in the manuscript and referred to as the Ornish diet dataset after the name of the conducted study, is a transcriptomics dataset ^6,7^.

## **OPERA dataset**

In particular, the second dataset - in terms of presenting them in the main paper – which is also referred to as the OPERA dataset after the name of the corresponding study^8^, has 4 individual binary labels. The distribution of the participants in these labels is shown in Supplementary Table 1.

# Python Libraries and versions

The Python libraries and their versions used in the implementation of the present manuscript is presented in Supplementary Table 3.

# Bioinformatics analysis

The results presented in the manuscript for the Ornish diet dataset were undergo a bioinformatics analysis to interpret their biological meaning. After the tissue/organ expression of the genes selected by the algorithm and the visualization co-expression network, an enrichment analysis was made to identify any biological terms or pathways enriched in the selected feature set. Pathway and Functional enrichment analysis was conducted using David Web-tool (<https://david.ncifcrf.gov/>) and a q-value threshold of 0.05 was used to infer significant terms.

# Random Forest optimized with Randomized Grid Search

For comparative reasons, we also trained and tested the Random Forest method using grid searching to optimize the model parameters. During this exercise grid searching using stratified 10-fold cross-validation was applied to test the following parameter values:

- 'n_estimators': randint(50, 1000)
- 'max_depth': [1, 2, 3, 4, 5, 6, 7, 8, 9, 10]
- 'min_samples_split:' [2, 3, 4, 5, 6, 7, 8, 9, 10]
- 'min_samples_leaf': [1, 2, 3, 4, 5, 6, 7, 8, 9, 10]

# Metrics

For the evaluation of solutions, multiple classical machine learning metrics for classification are used. Among these metrics, two custom model complexity measures were also implemented to measure the number of features in each model and the number of splits in each learner of the ensemble of the XGBoost model.

Model’s complexity (features):

Custom metric coded in Python.

$Complexity_{features}= \frac{a}{a+\#selected features}$ (1)

Model’s complexity (splits):

Custom metric coded in Python.

$Complexity_{splits}= 1-\frac{number of splits}{\max\left( allowed depth \right)*2^{\left( \max\left( depth \right)-1 \right)}}$ (2)

Accuracy:

Sklearn package of python

$Accuracy= \frac{TP+TN}{TP+TN+FP+FN}$ (3)

Weighted geometric mean (wGM):

Custom metric coded in Python.

$wGM= \frac{1}{N}\sum_{i\in I} \left( \sqrt{Sensitivity\cdot Specificity} \cdot support_{i} \right)=\frac{1}{N}\sum_{i\in I} \left( \sqrt{\frac{TP}{TP+FN}\cdot\frac{TN}{TN+FP}} \cdot support_{i} \right)$ (4)

Balanced Accuracy (bAcc):

Sklearn package of python (balanced_accuracy_score())

$bAcc=\frac{Sensitivity+Specificity}{2}=\frac{\frac{\boldsymbol{TP}}{\boldsymbol{TP + FN}} + \frac{\mathrm{TN}}{TN + FP}}{2}$ (5)

Precision

Sklearn package of python (precision_recall_fscore_support(average=avg,zero_division = 0)[0])

$Precision =\frac{TP}{TP+FP}$ (6)

Recall

Sklearn package of python (precision_recall_fscore_support(average=avg,zero_division = 0)[1])

$Recall =\frac{TP}{TP+FN}$ (7)

F1 Score

Sklearn package of python (precision_recall_fscore_support(average=avg,zero_division = 0)[2])

$F1 =2\cdot\frac{Precision \cdot Recall}{Precision + Recall}$ (8)

F2 Score

Sklearn package of python (fbeta_score(beta=2))

ROC_AUC

Sklearn package of python (roc_auc_score(average = avg))

# Algorithm

1. data preprocessing
2. pseudo-random initialization of solutions
3. **While** max_num_generations not reached AND Similarity_ending_criterion not reached:
4. **for** k 🡨 0,10:
5. train of N models
6. Evaluation of model on the selected metrics
7. ‘Best’ individual pass to the next generation
8. Pareto frontier ranking of solutions.
9. Nitch degradation of similar solutions (genome closeness) in the same Pareto front
10. Selection of N-1 solutions (randomly, non-uniformly) to pass to the next generation
11. crossover on pairs of solutions (90% chance)
12. Mutation of single solutions on feature- and or parameter-genes (5% chance)
13. Calculate the similarity of solutions
14. **for** k 🡨 0,10:
15. train of N models
16. Evaluation of the model on the selected metrics
17. Pareto frontier ranking of solutions
18. Save all models of the 1^st^ Pareto frontier

# Supplementary Figures


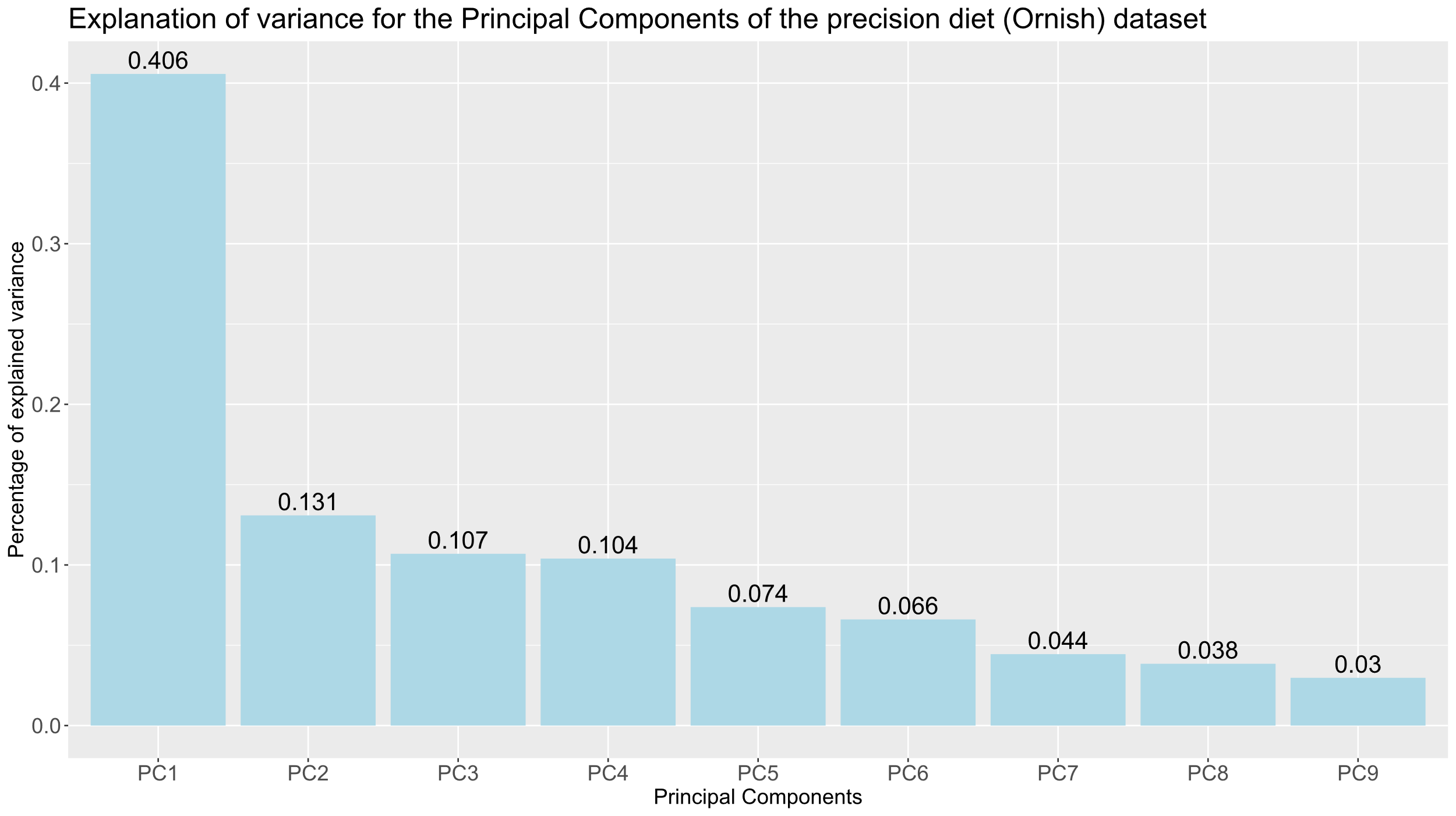


Supplementary Figure. 1. Principal Components loadings (variance explanation) for the Ornish diet dataset


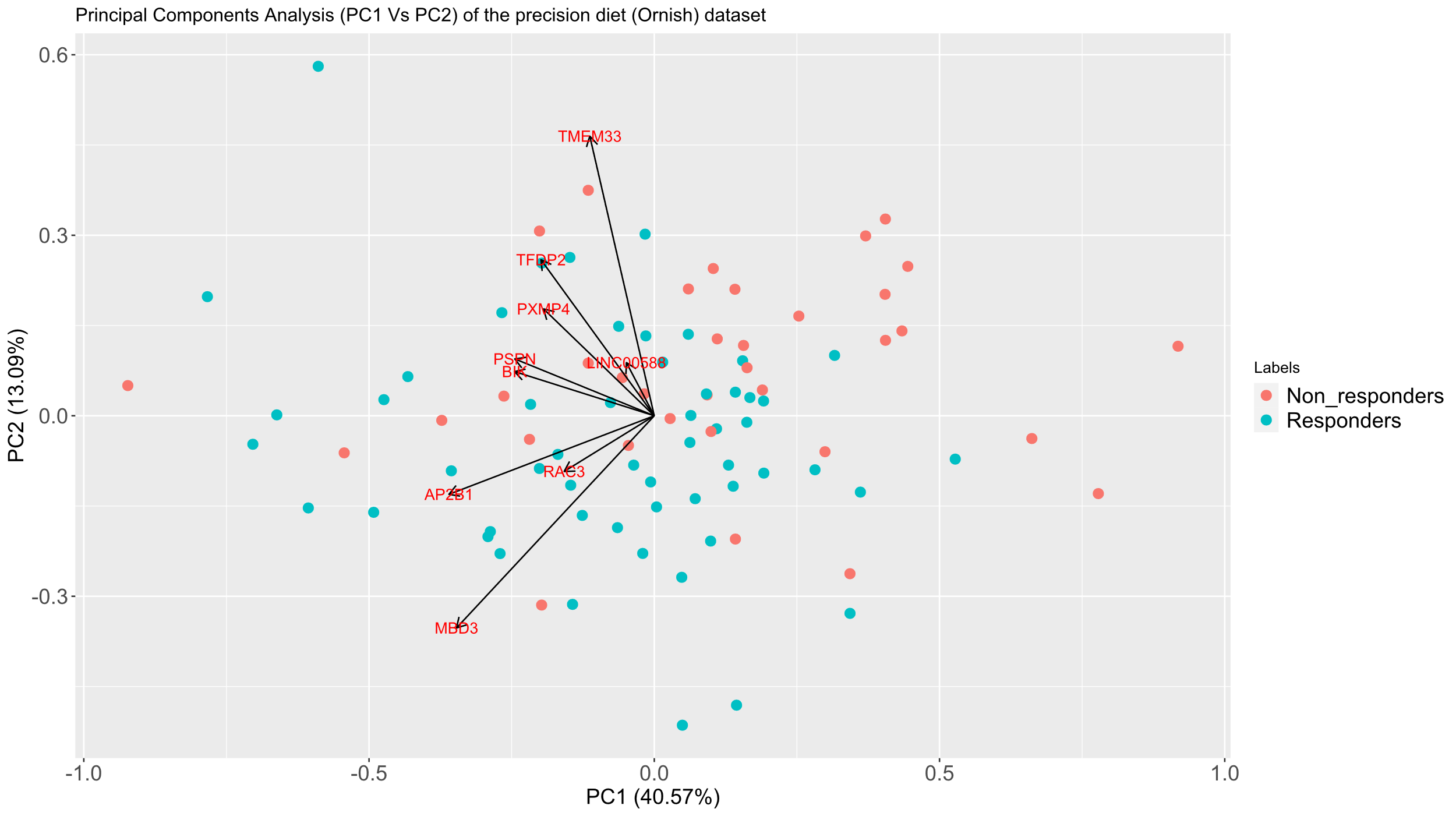


Supplementary Figure. 2. Principal Component Analysis (PCA) visualization of components with the highest loadings (PC1 and PC2) for the Ornish diet dataset


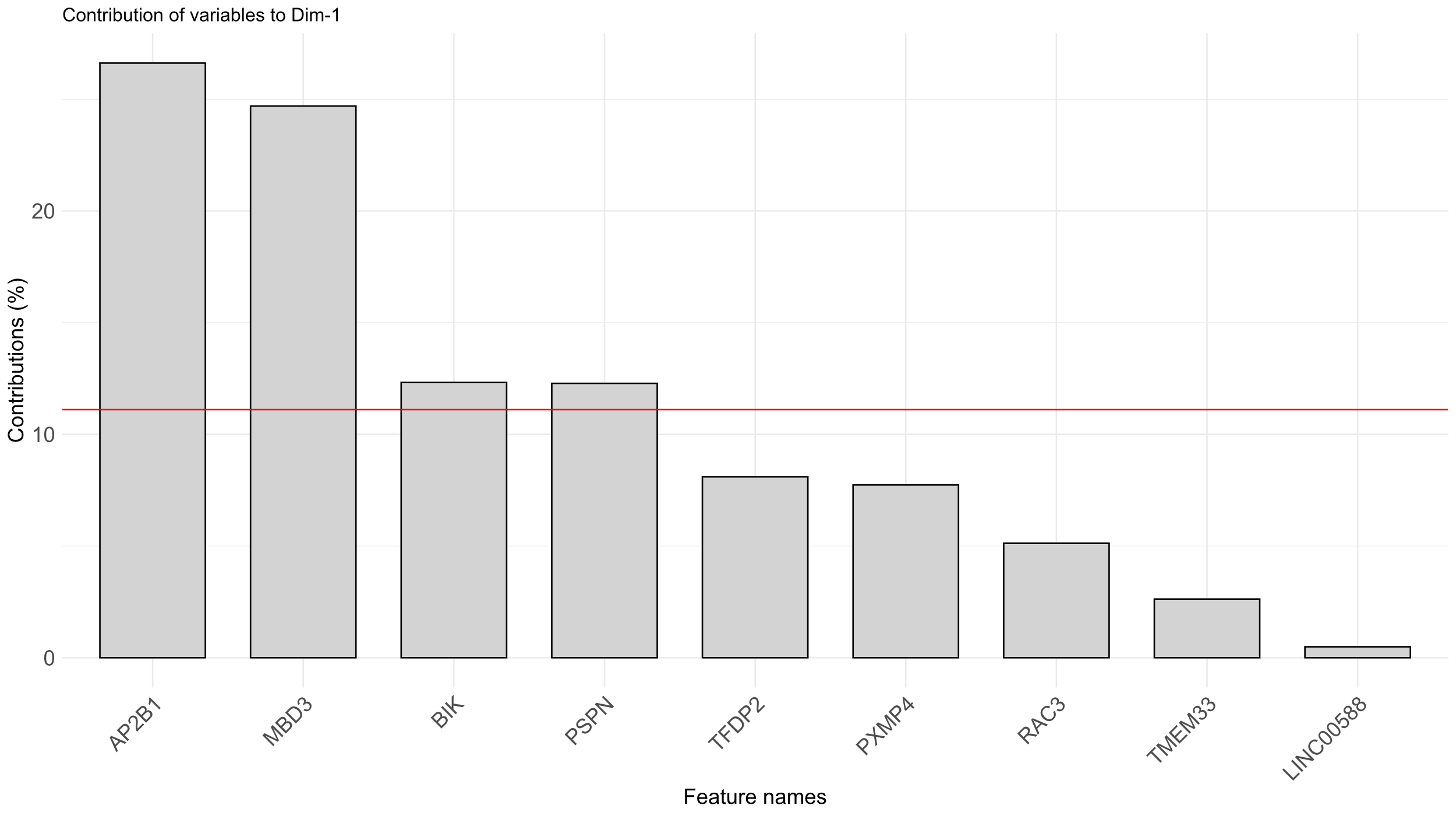


Supplementary Figure. 3. Contribution of the selected by MEvA-X features in the PC1 of the Ornish diet dataset


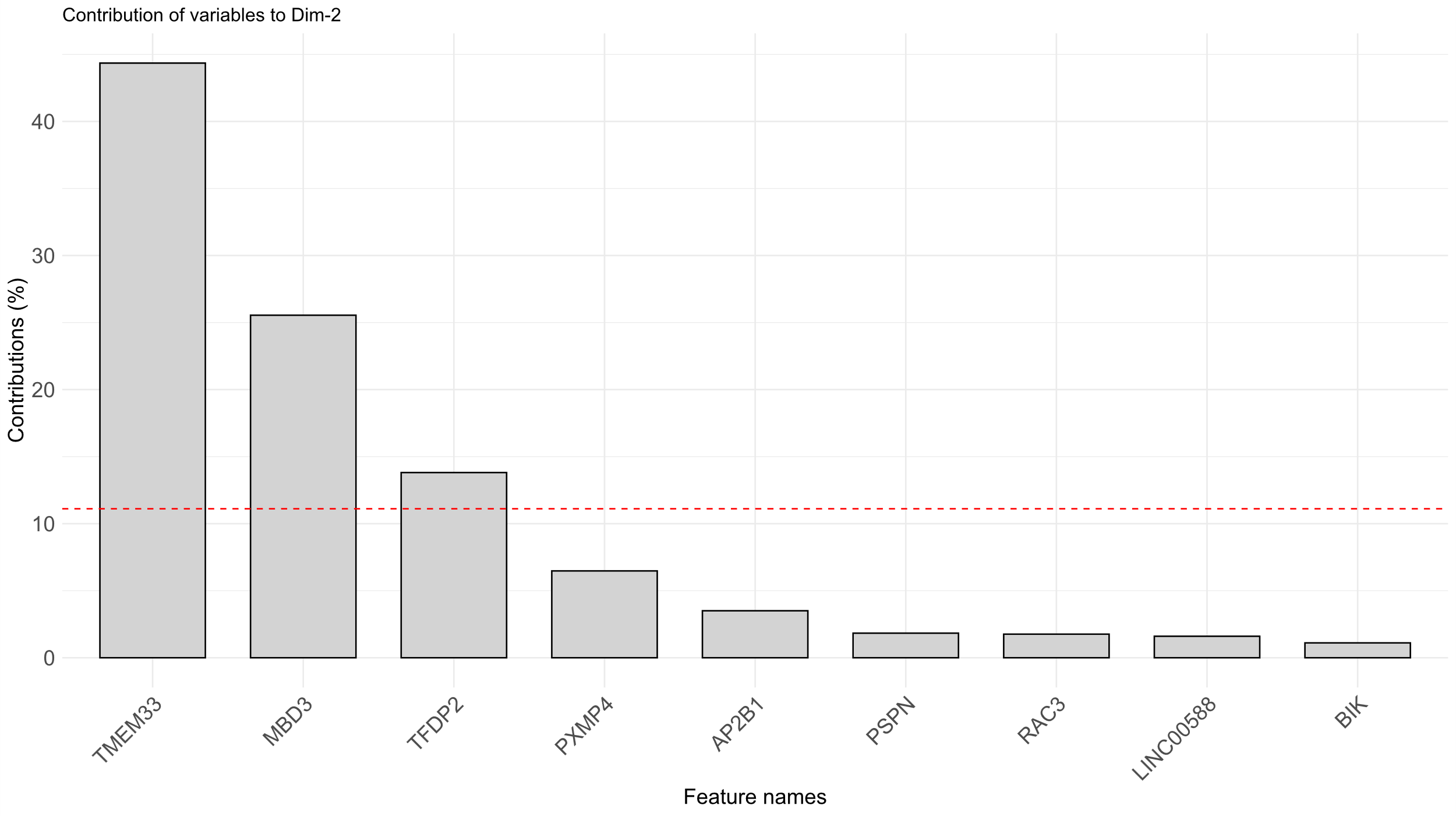


Supplementary Figure. 4. Contribution of the selected by MEvA-X features in the PC2 of the Ornish diet dataset


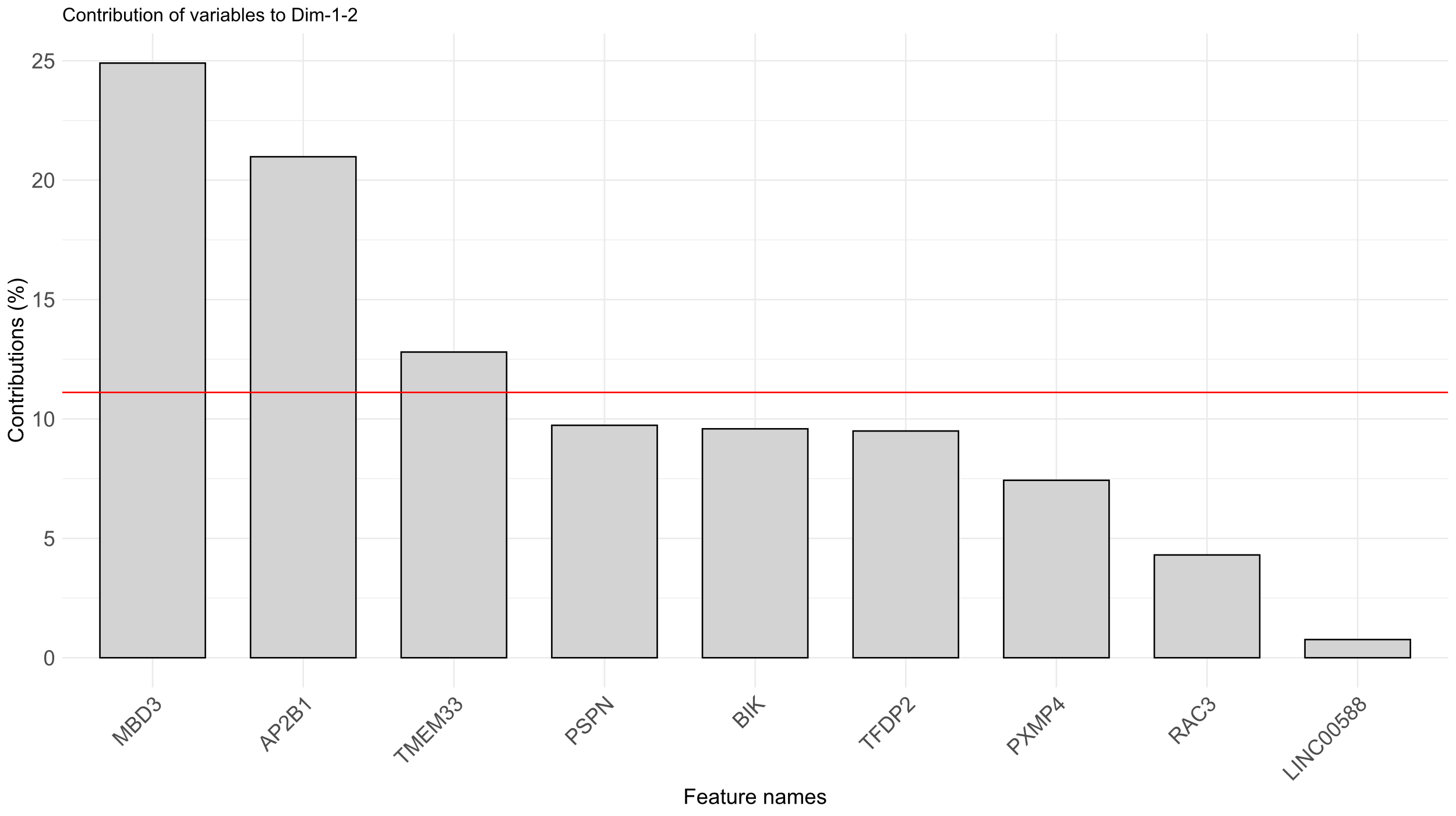
 Supplementary Figure. 5. Contribution of the selected by MEvA-X features in the PC1&PC2 of the Ornish diet dataset


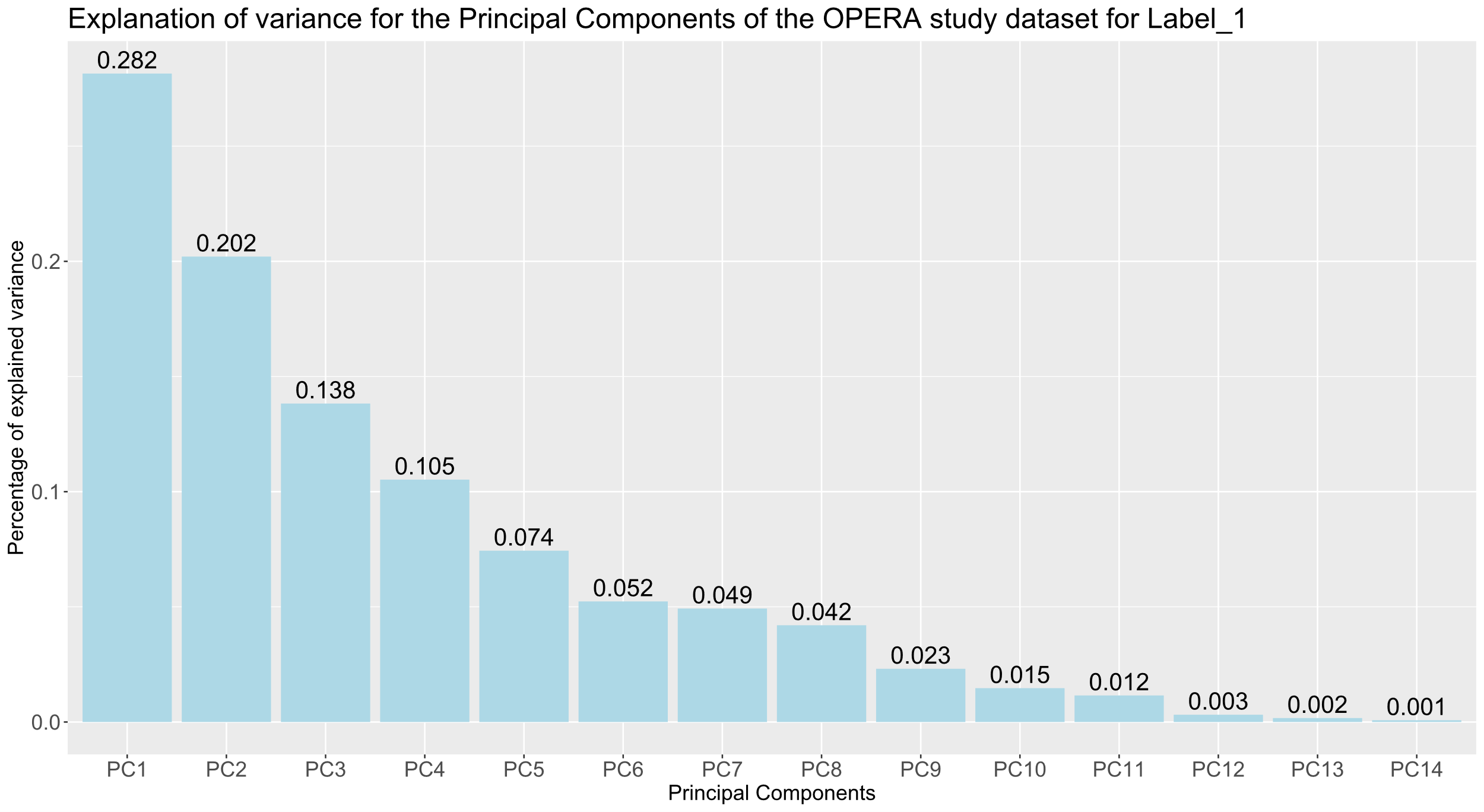
*Supplementary Figure*. 6. Principal Components loadings (variance explanation) for the OPERA dataset for the Severity_Change Label (Label_1)


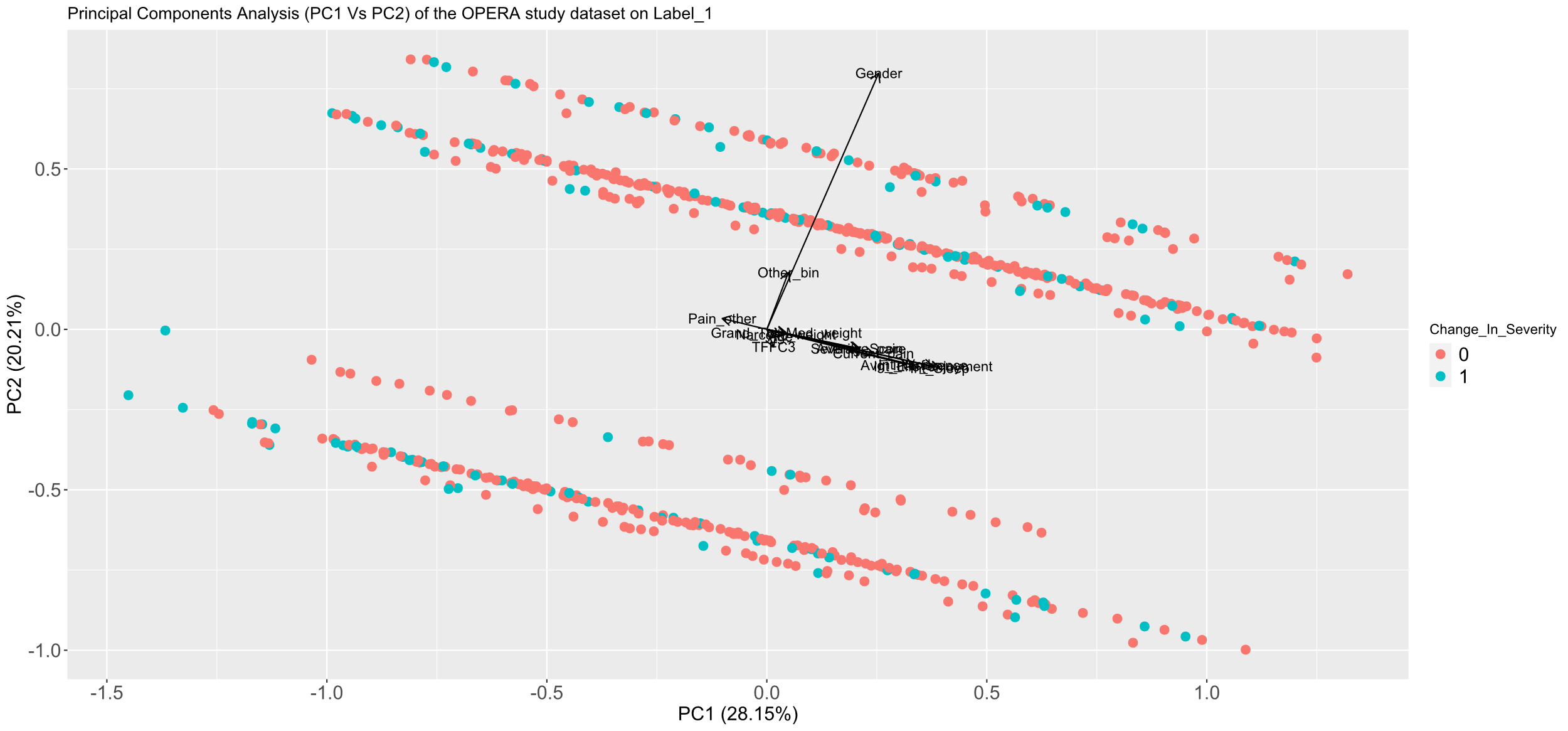


Supplementary Figure. 7. Principal Component Analysis (PCA) visualization of components with the highest loadings (PC1 and PC2) for the Severity_Change Label (Label_1)


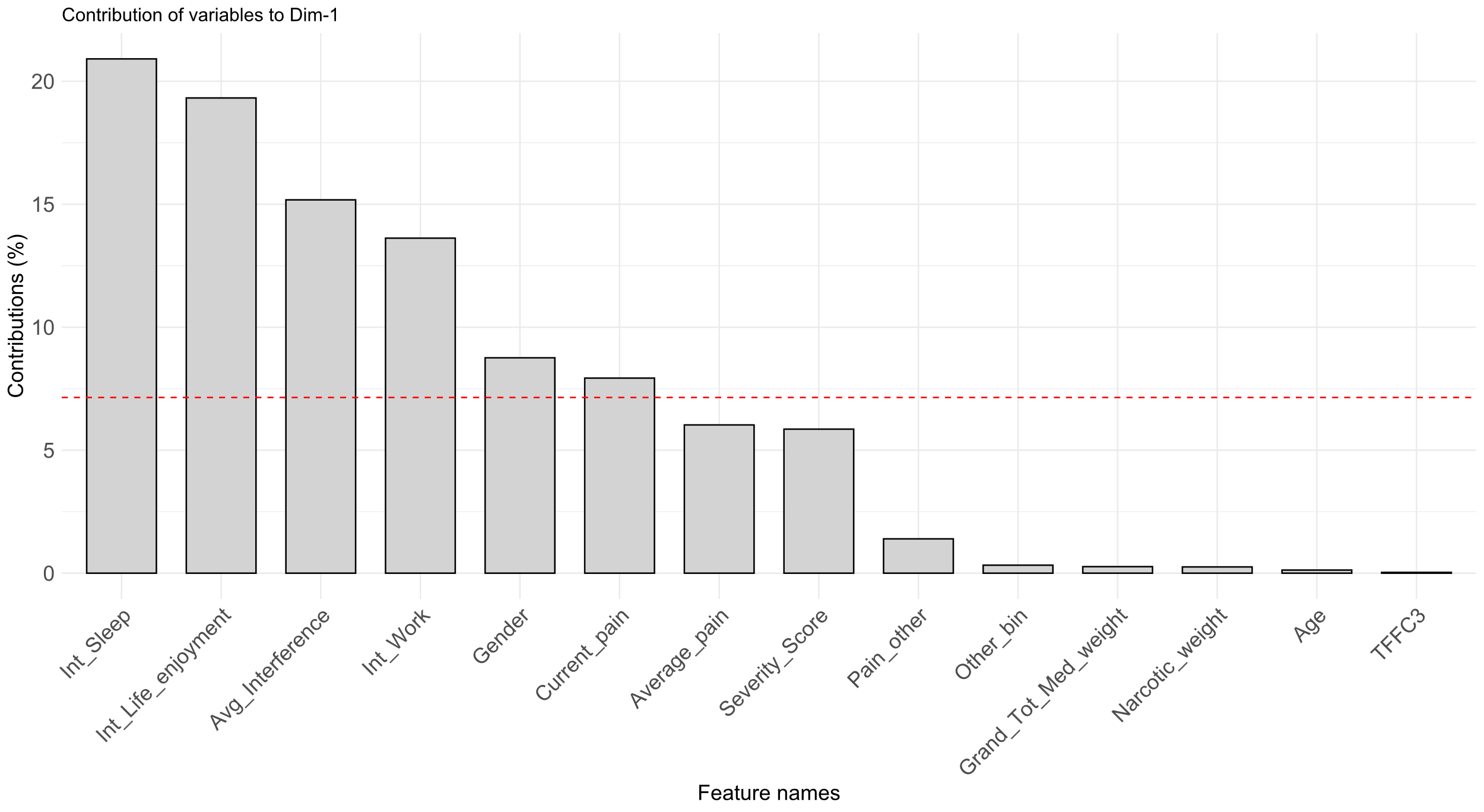


Supplementary Figure. 8. Contribution of the selected by MEvA-X features in the PC1 of the OPERA dataset, Label_1 (Severity_Change Label)


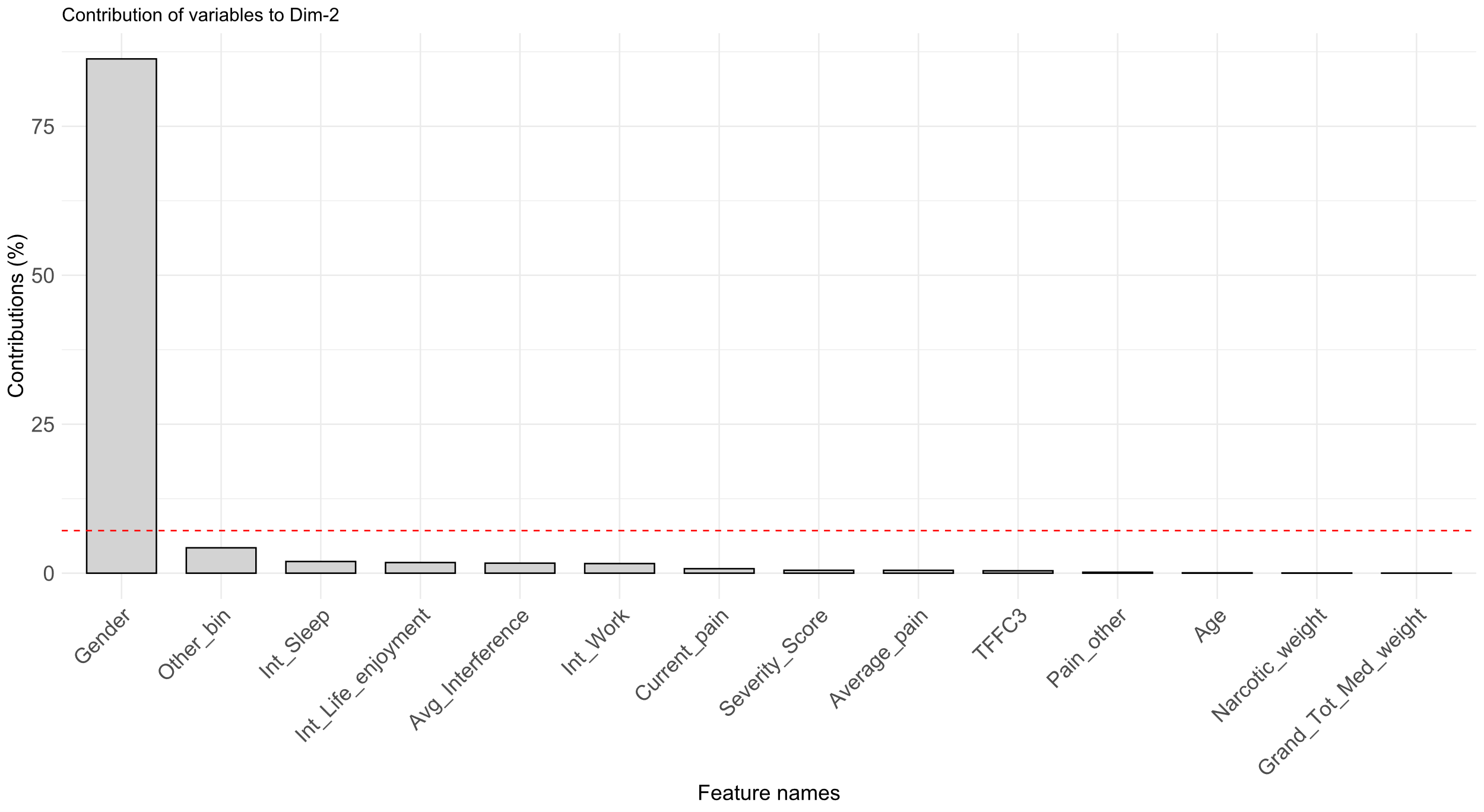


Supplementary Figure. 9. Contribution of the selected by MEvA-X features in the PC2 of the OPERA dataset, Label_1 (Severity_Change Label)


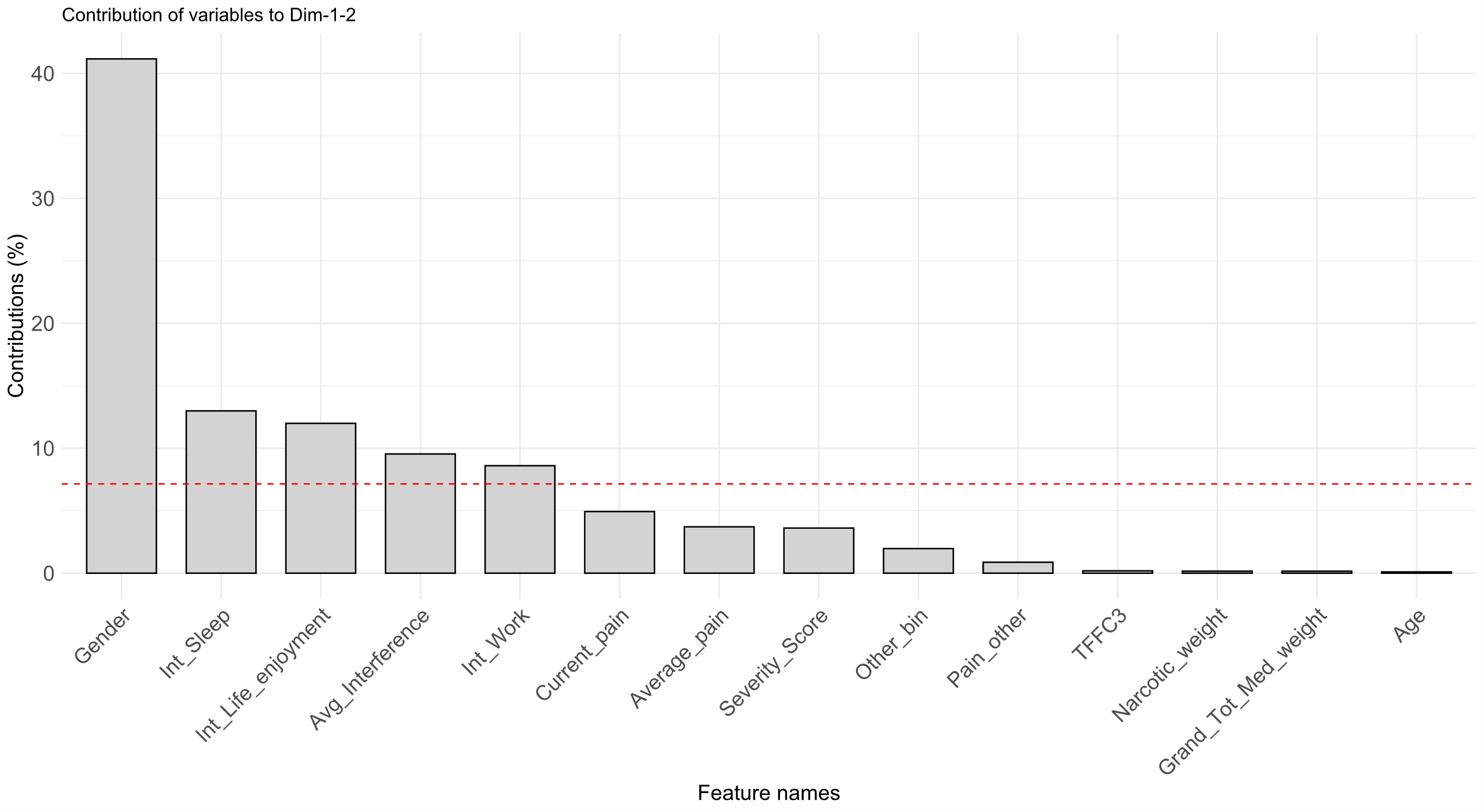


Supplementary Figure. 10. Contribution of the selected by MEvA-X features in the PC1&PC2 of the OPERA dataset, Label_1 (Severity_Change Label)


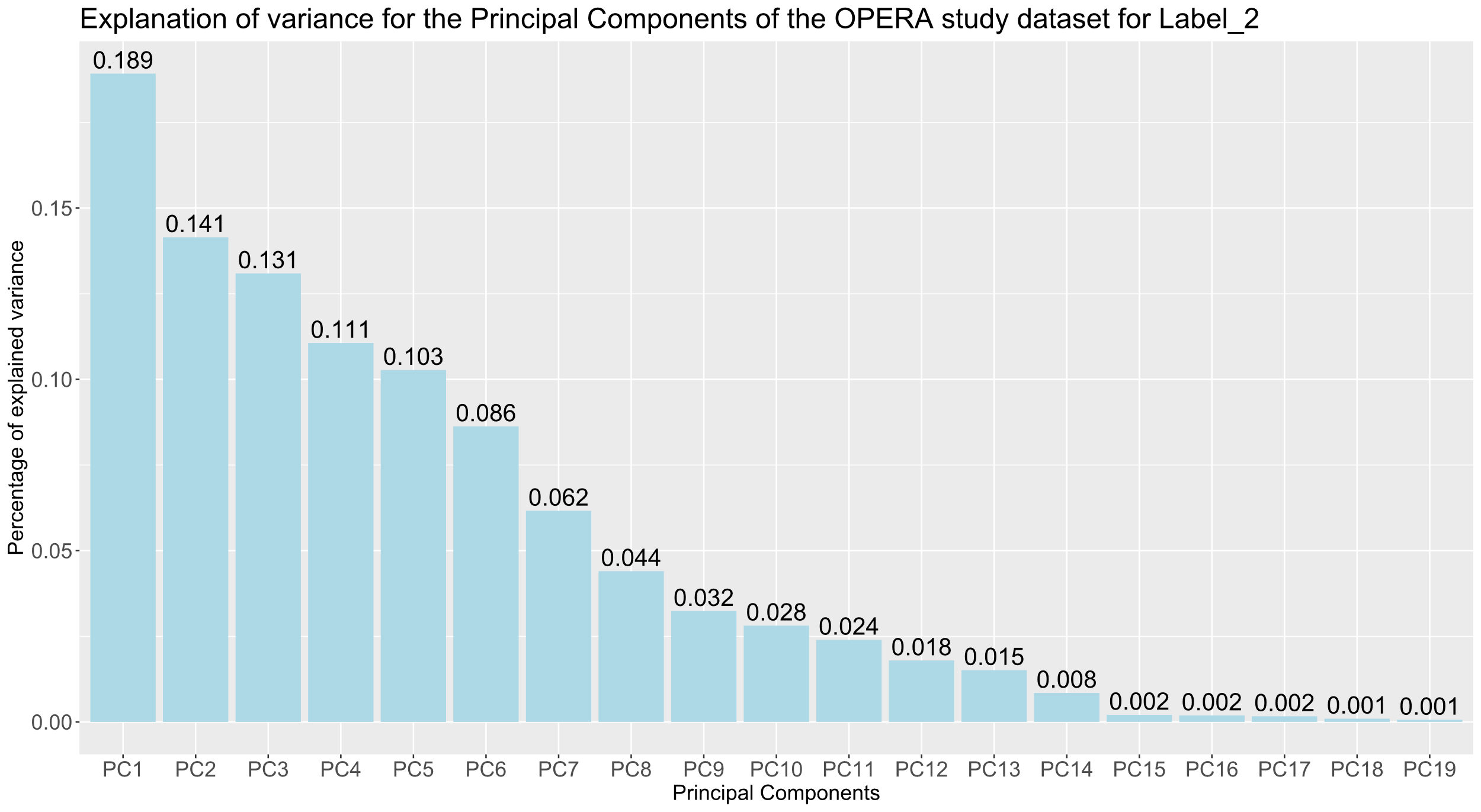


Supplementary Figure. 11. Principal Components loadings (variance explanation) for the OPERA diet dataset for the Label_2


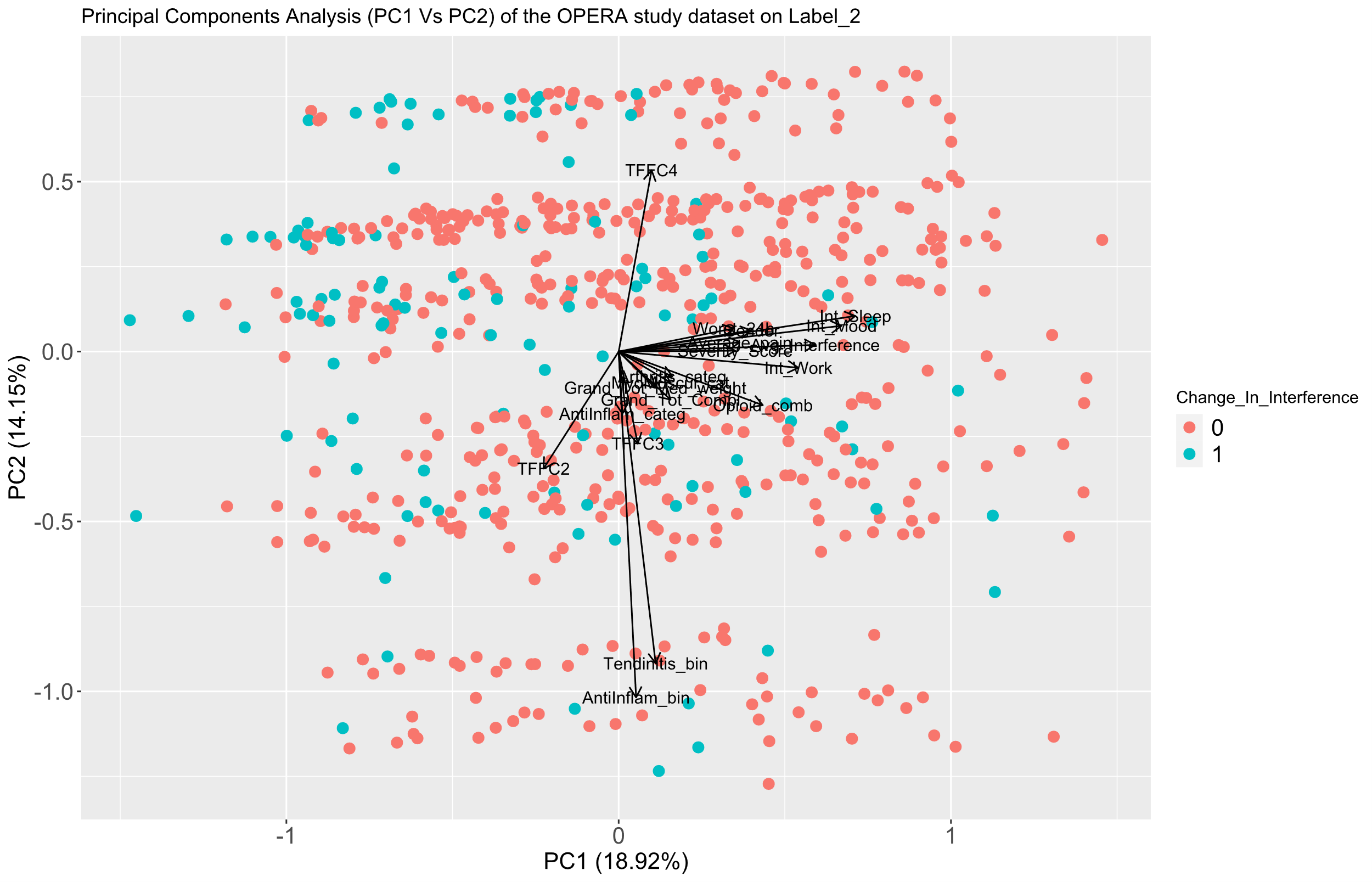


Supplementary Figure. 12. Principal Component Analysis (PCA) visualization of components with the highest loadings (PC1 and PC2) for the Interference_Change Label (Label_2)


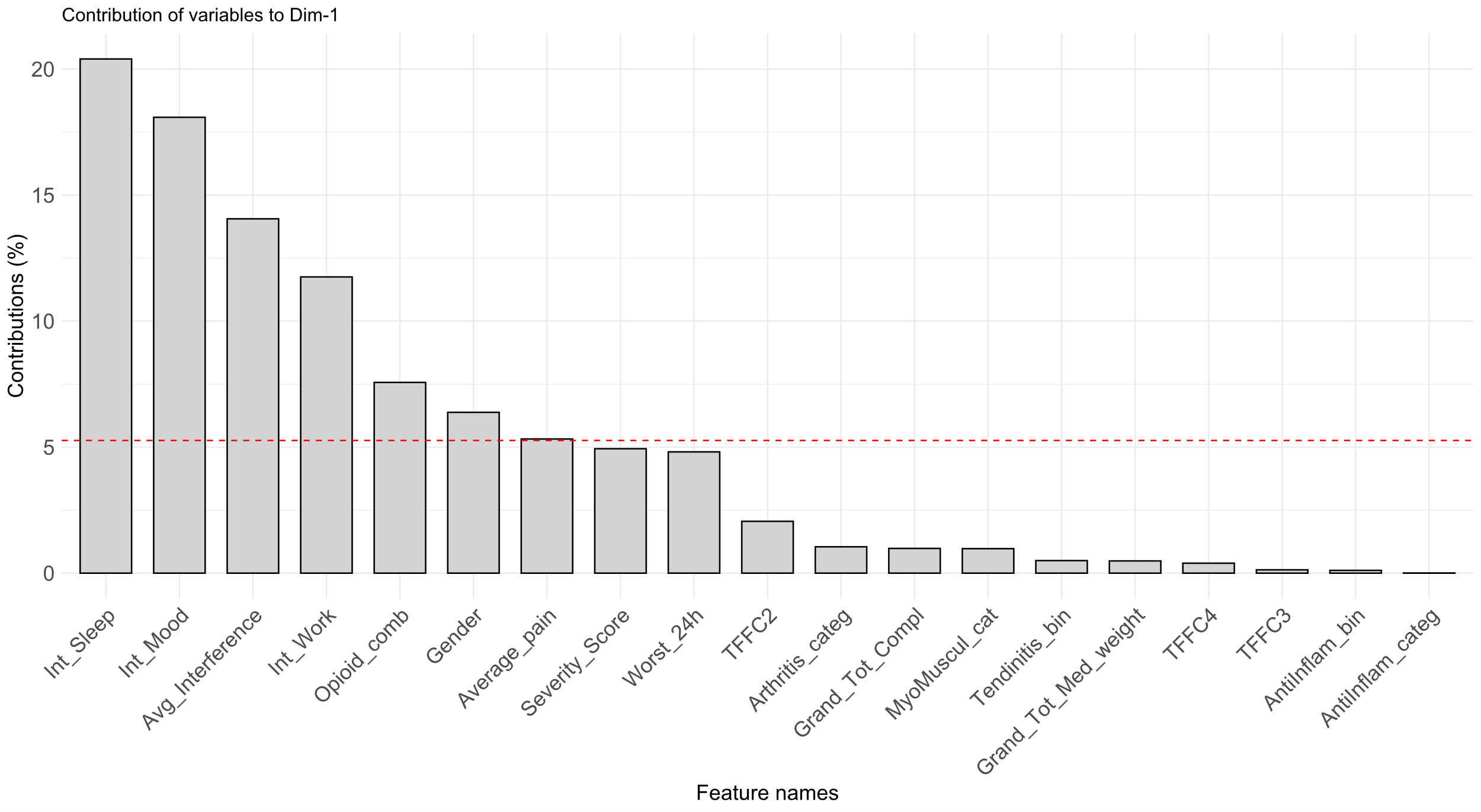


Supplementary Figure. 13. Contribution of the selected by MEvA-X features in the PC1 of the OPERA dataset, Label_2 (Interference_Change Label)


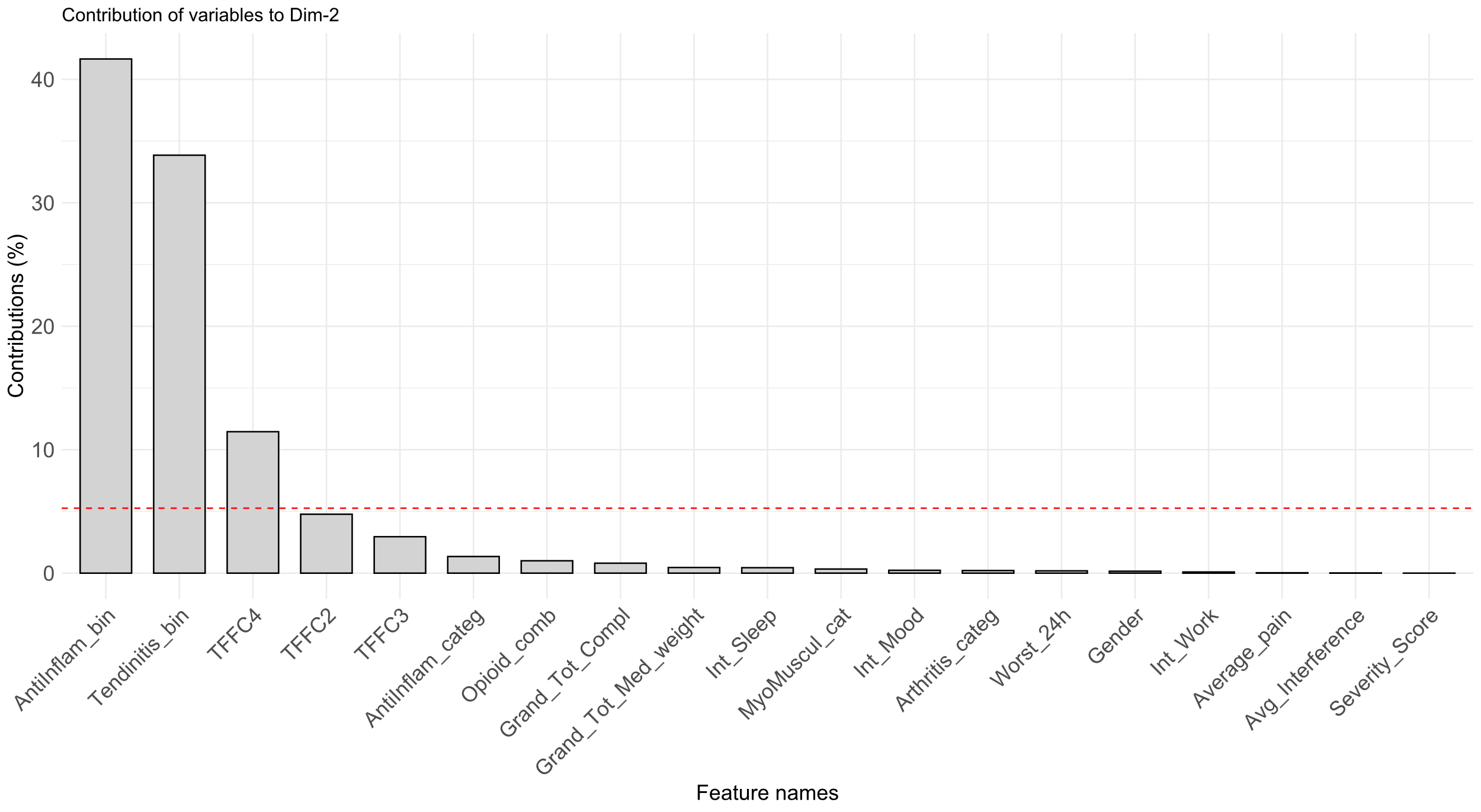


Supplementary Figure 14. Contribution of the selected by MEvA-X features in the PC2 of the OPERA dataset, Label_2 (Interference_Change Label)


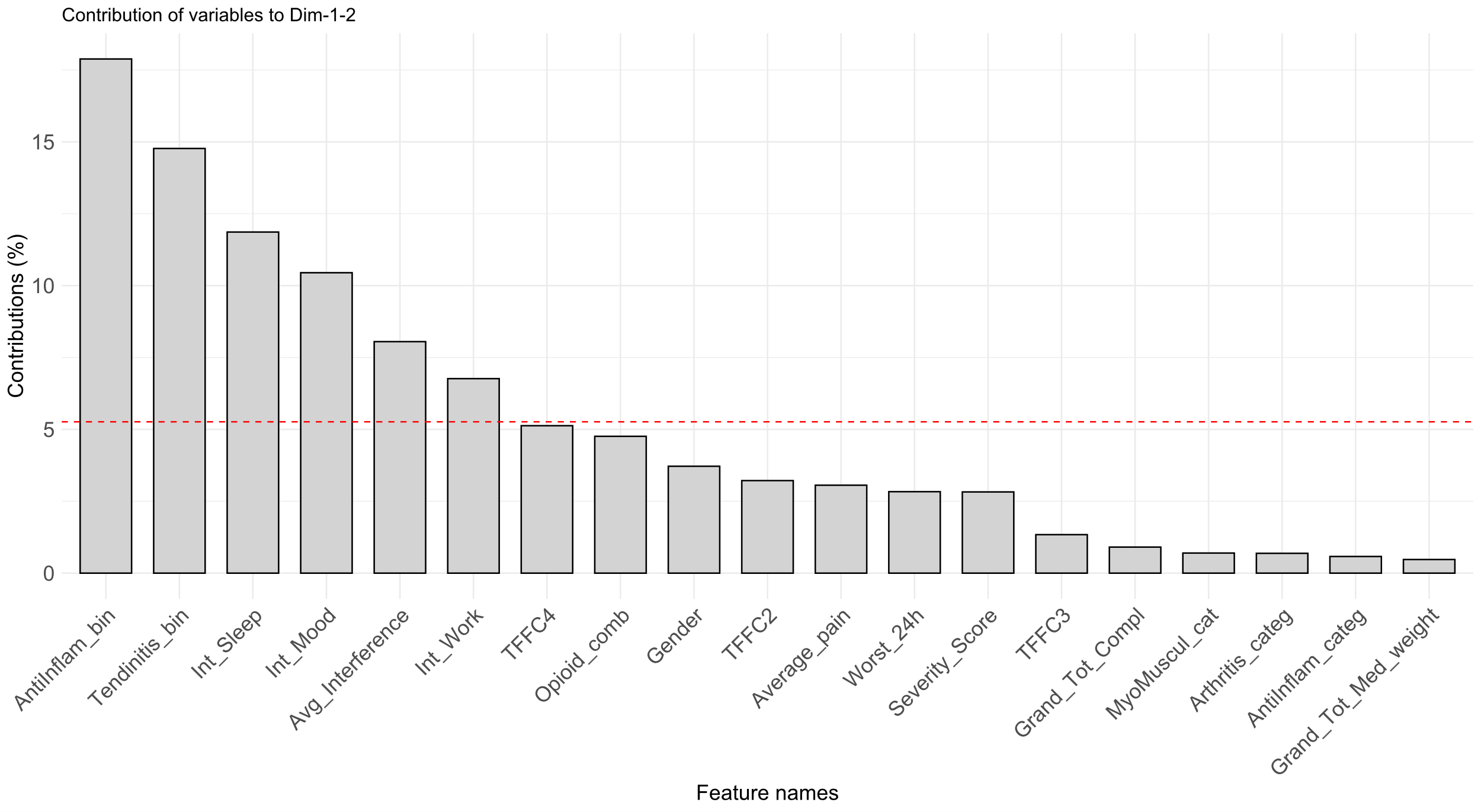


Supplementary Figure 15. Contribution of the selected by MEvA-X features in the PC1&PC2 of the OPERA dataset, Label_2 (Interference_Change Label)


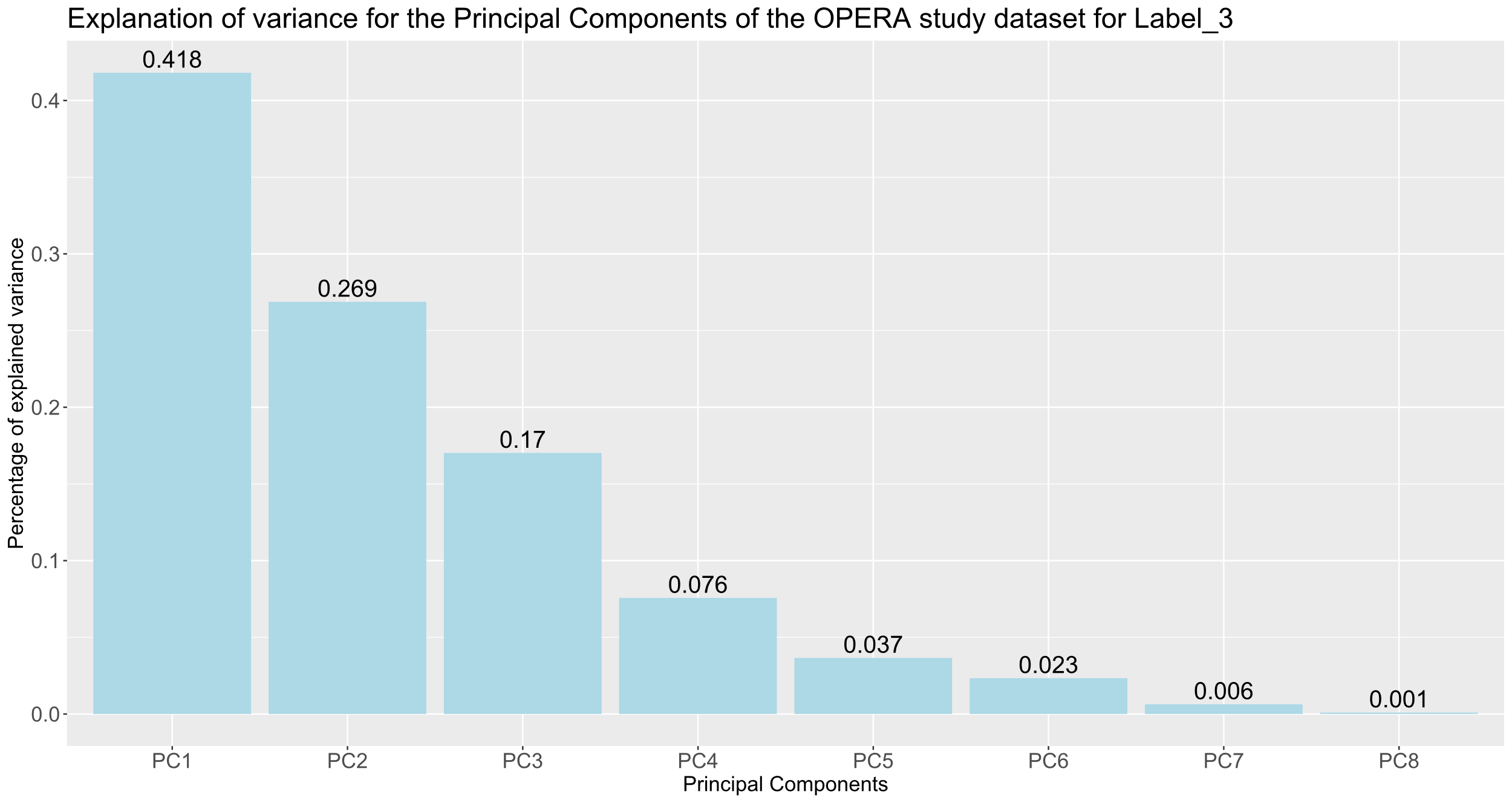


Supplementary Figure 16. Principal Components loadings (variance explanation) for the OPERA diet dataset for the Label_3


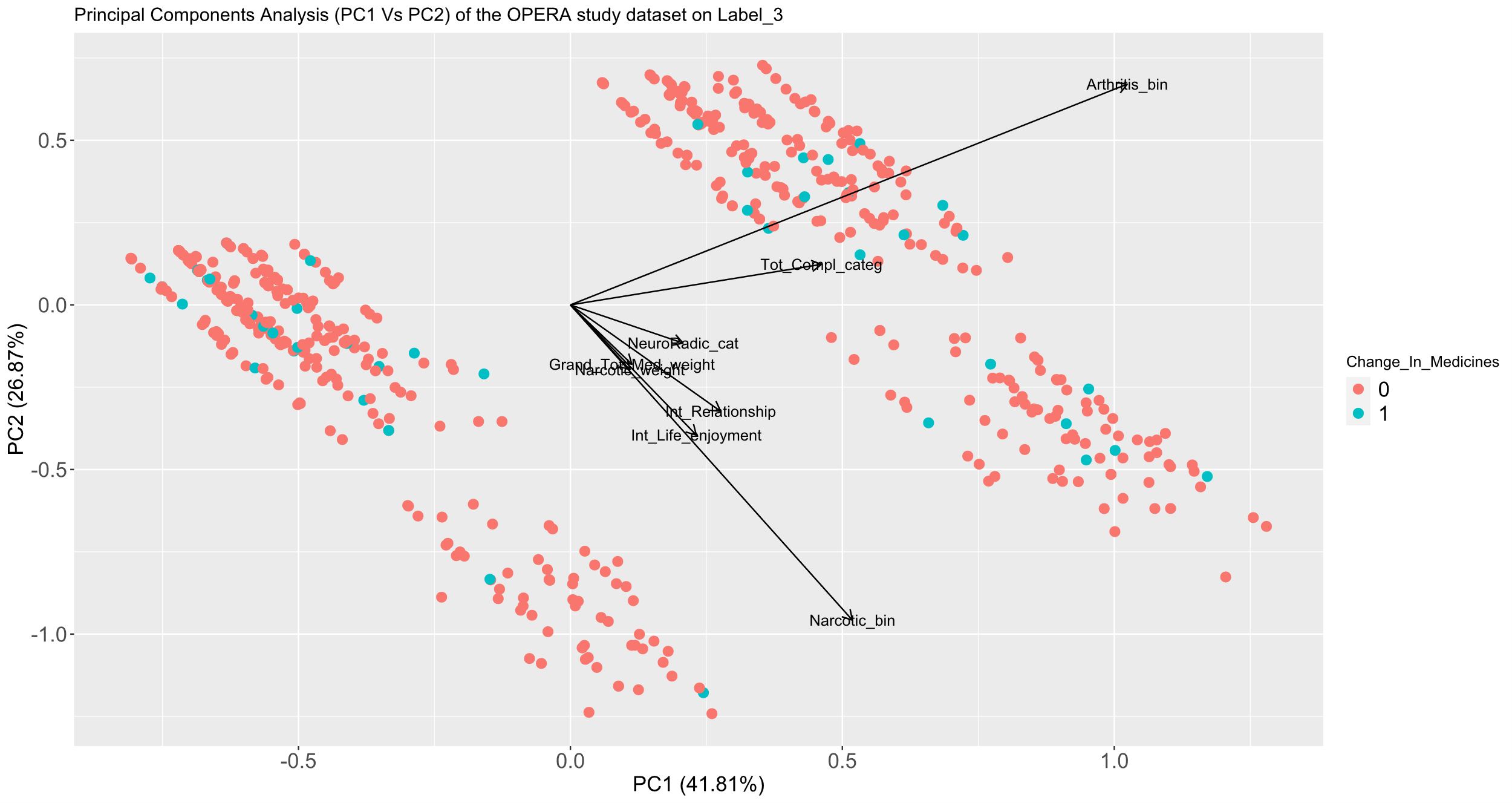


Supplementary Figure 17. Principal Component Analysis (PCA) visualization of components with the highest loadings (PC1 and PC2) for the Total_Drug_Change Label (Label_3)


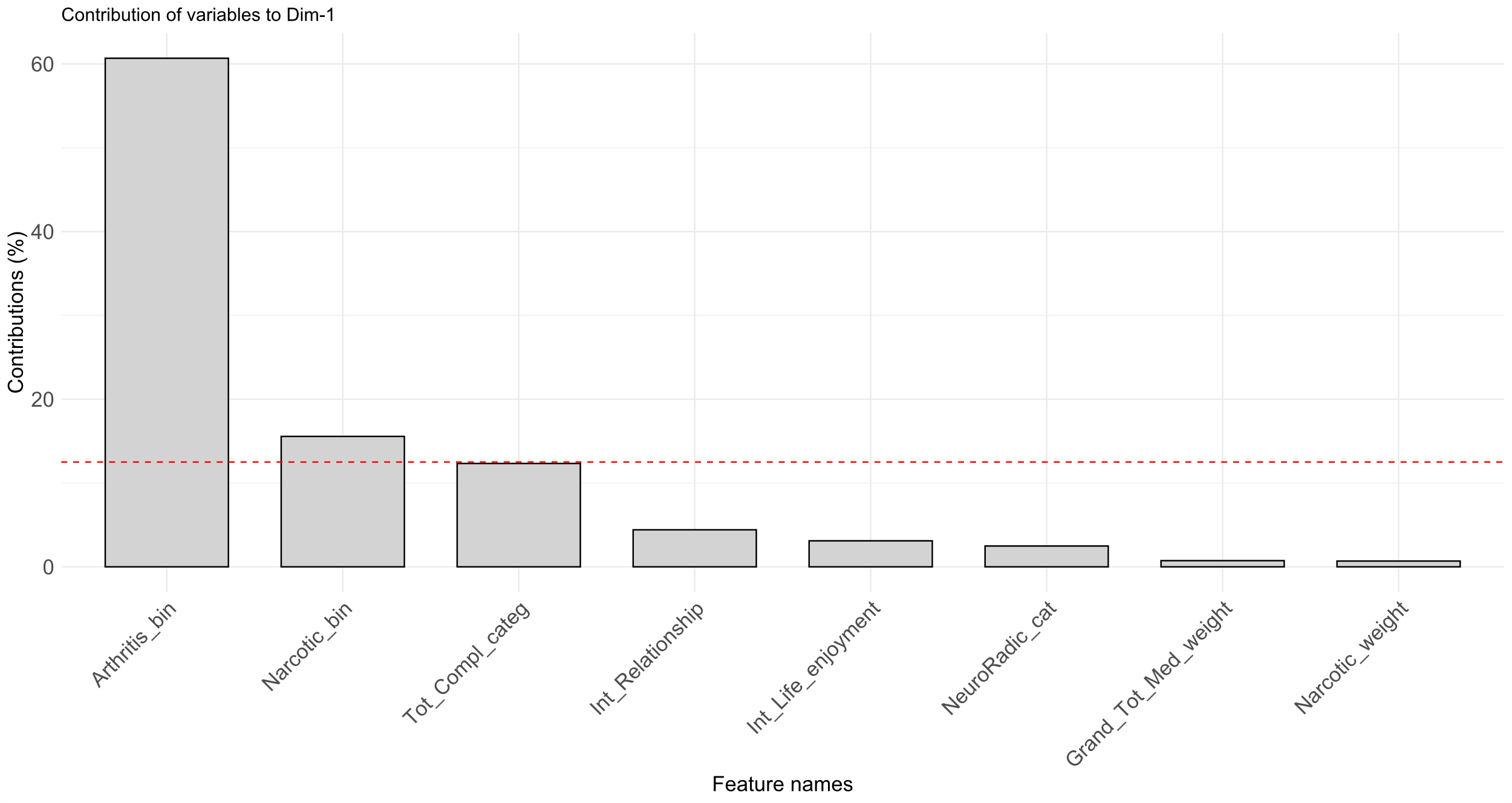


Supplementary Figure 18. Contribution of the selected by MEvA-X features in the PC1 of the OPERA dataset, Label_3 (Total_Drug_Change Label)


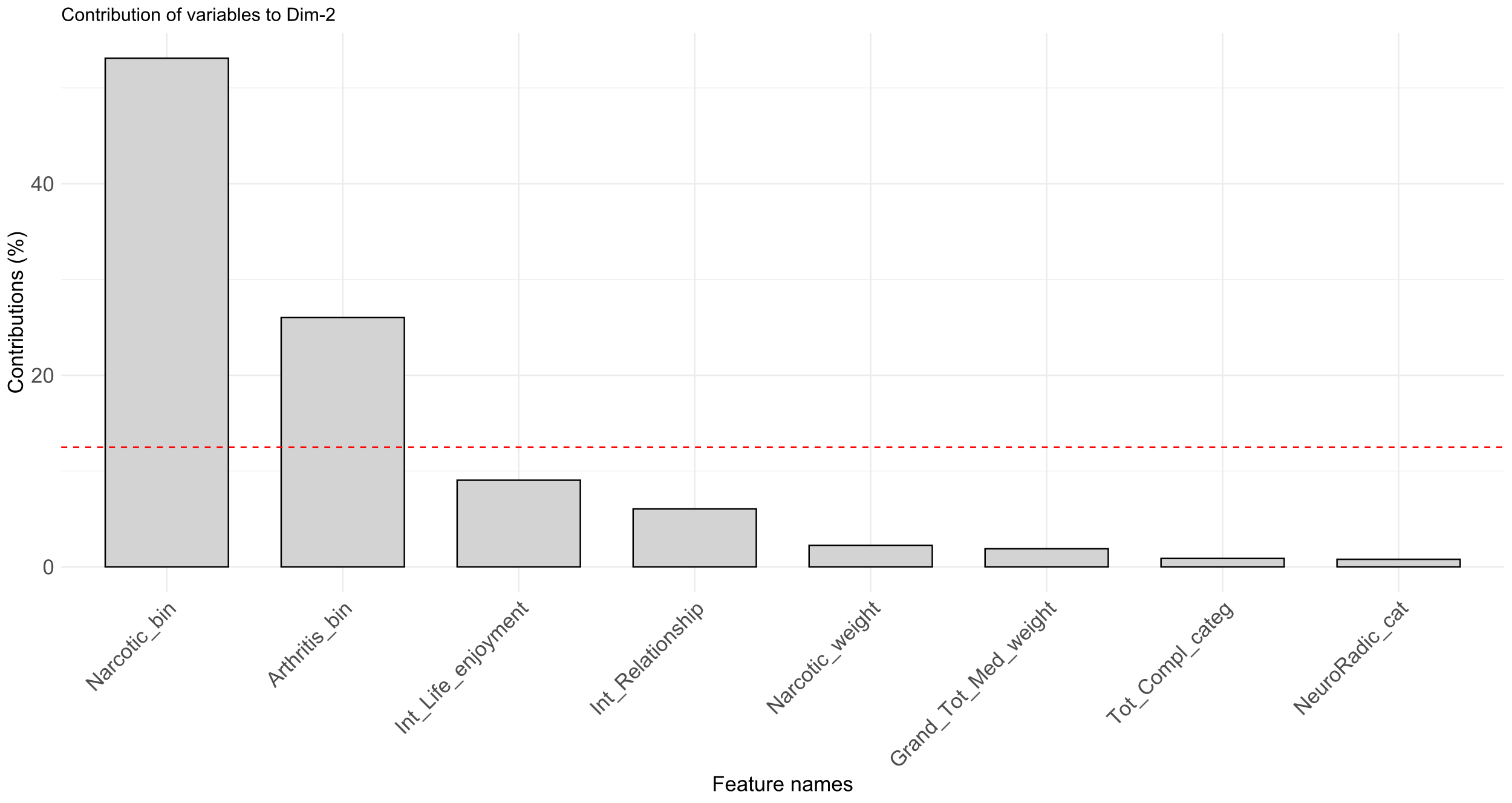


Supplementary Figure 19. Contribution of the selected by MEvA-X features in the PC2 of the OPERA dataset, Label_3 (Total_Drug_Change Label)


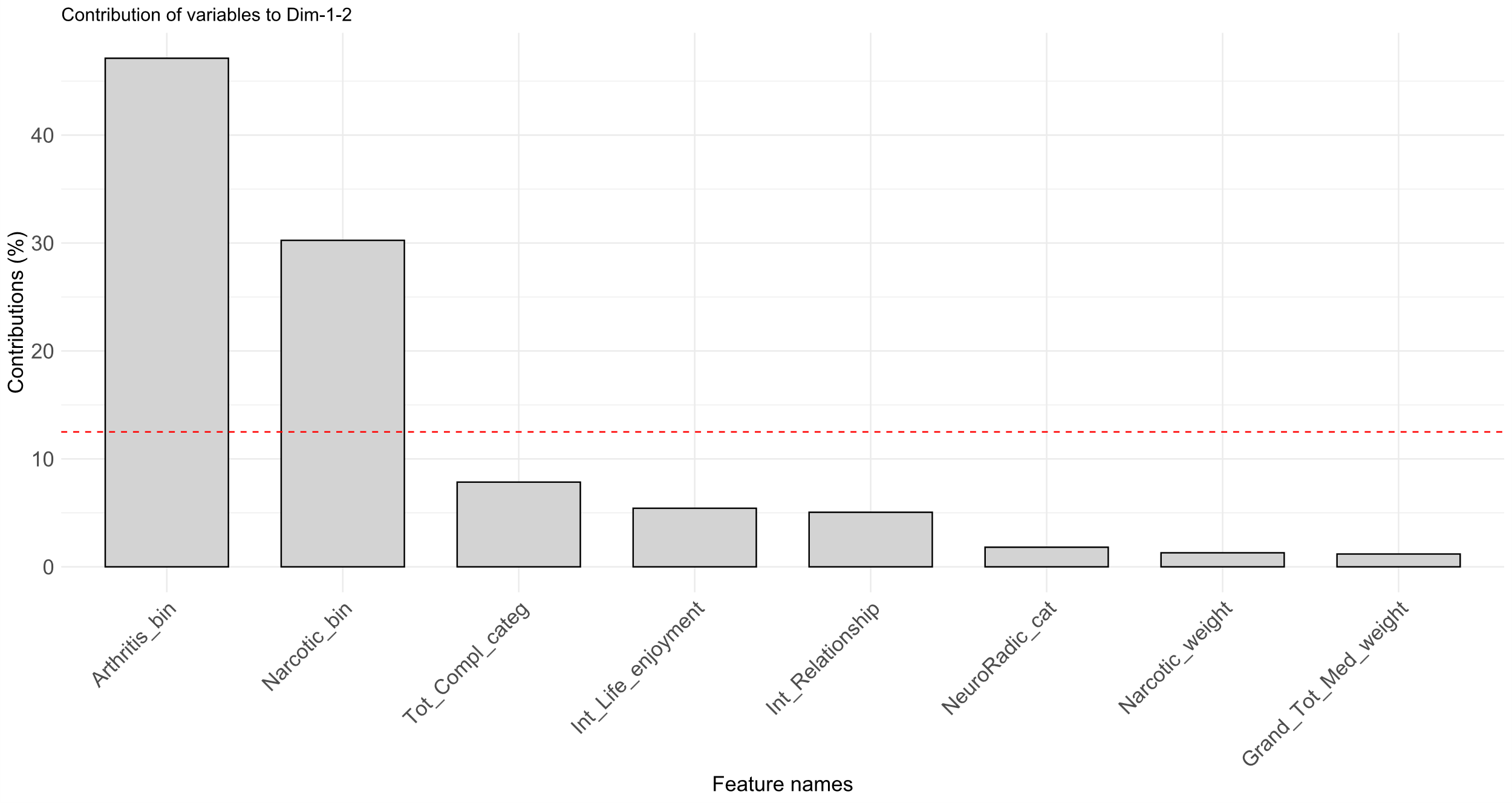


Supplementary Figure 20. Contribution of the selected by MEvA-X features in the PC1&PC2 of the OPERA dataset, Label_3 (Total_Drug_Change Label)


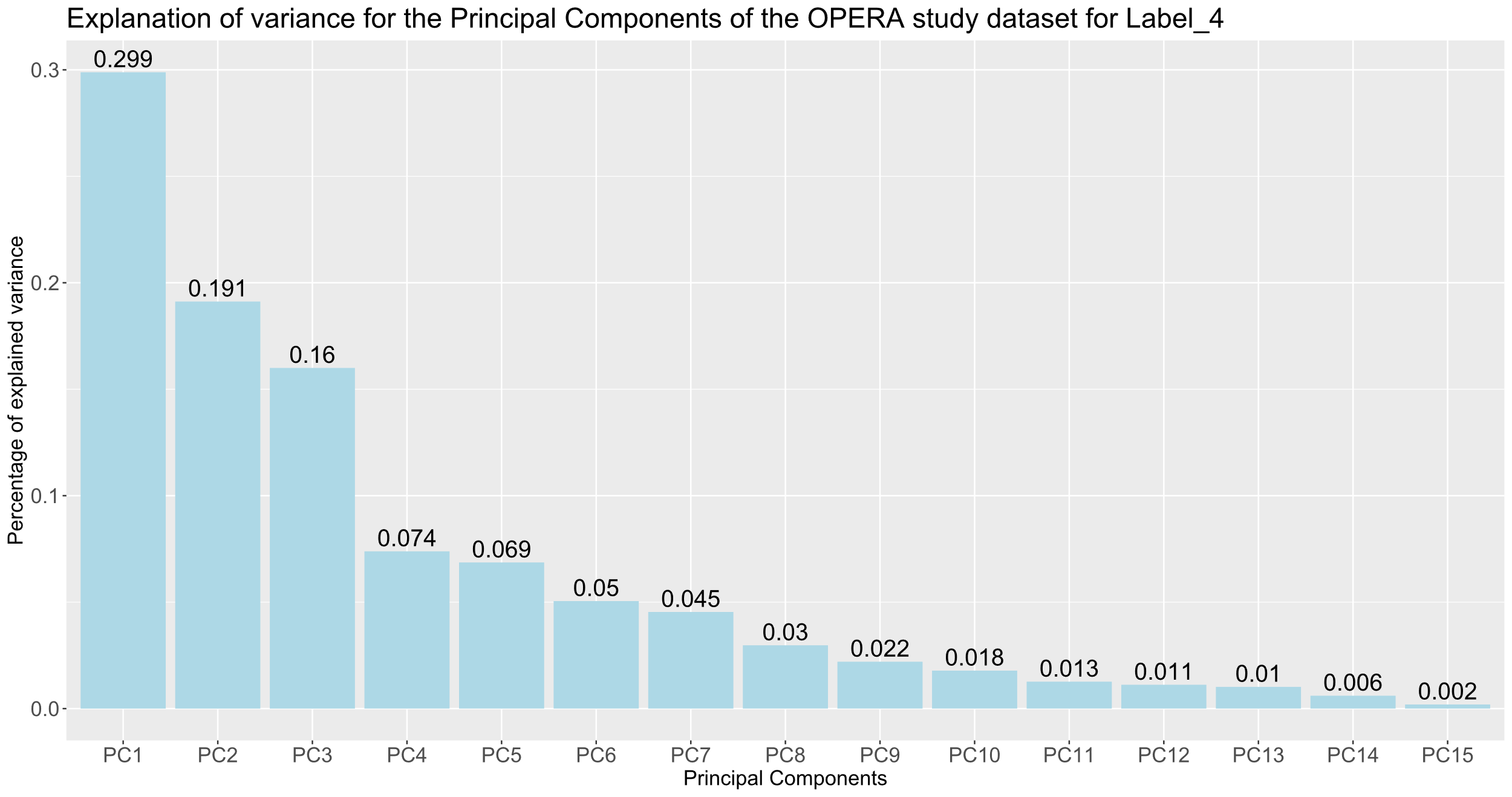


Supplementary Figure 21. Principal Components loadings (variance explanation) for the OPERA diet dataset for the Label_4


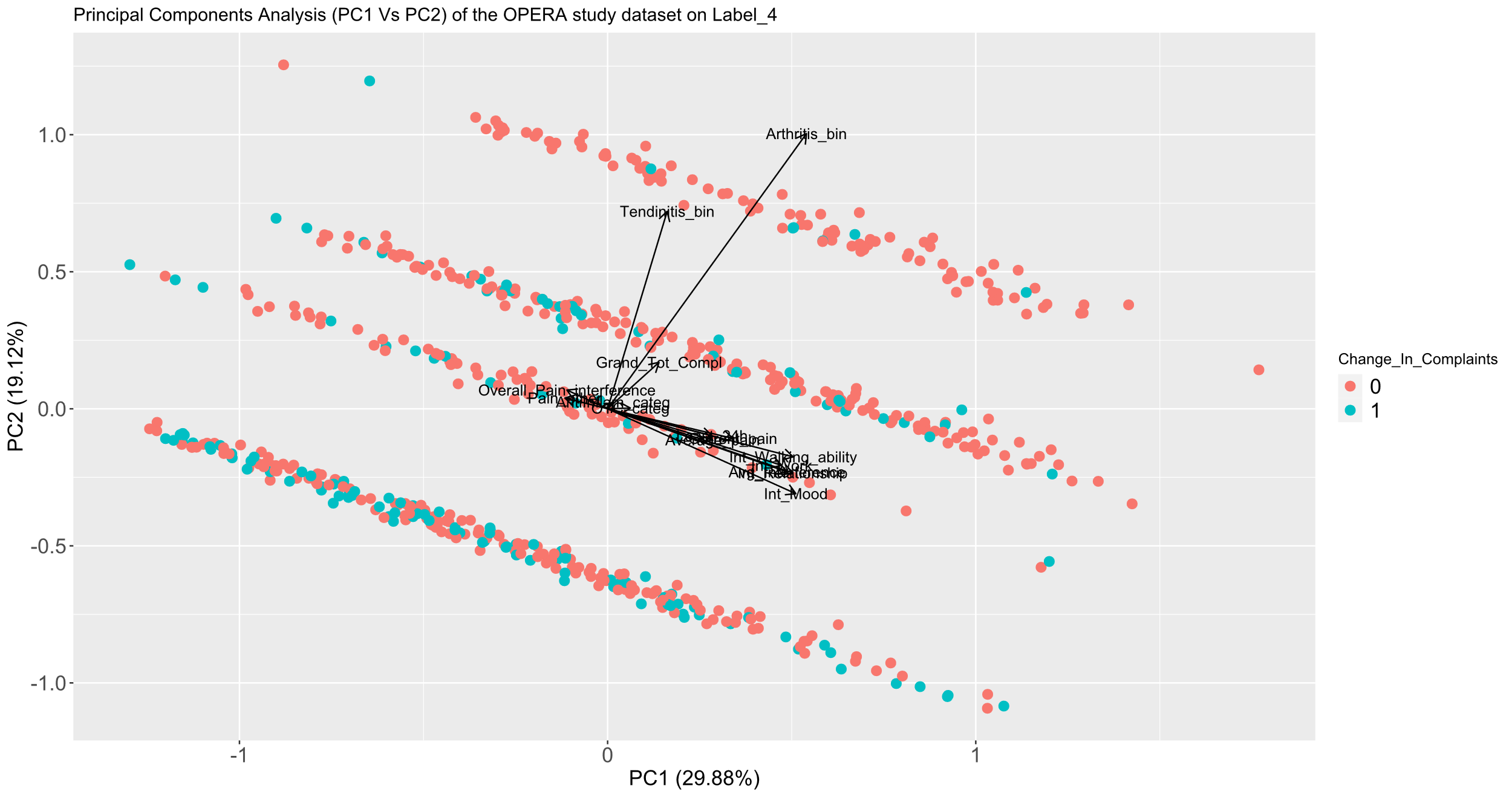


Supplementary Figure 22. Principal Component Analysis (PCA) visualization of components with the highest loadings (PC1 and PC2) for the Complaints_Change Label (Label_4)


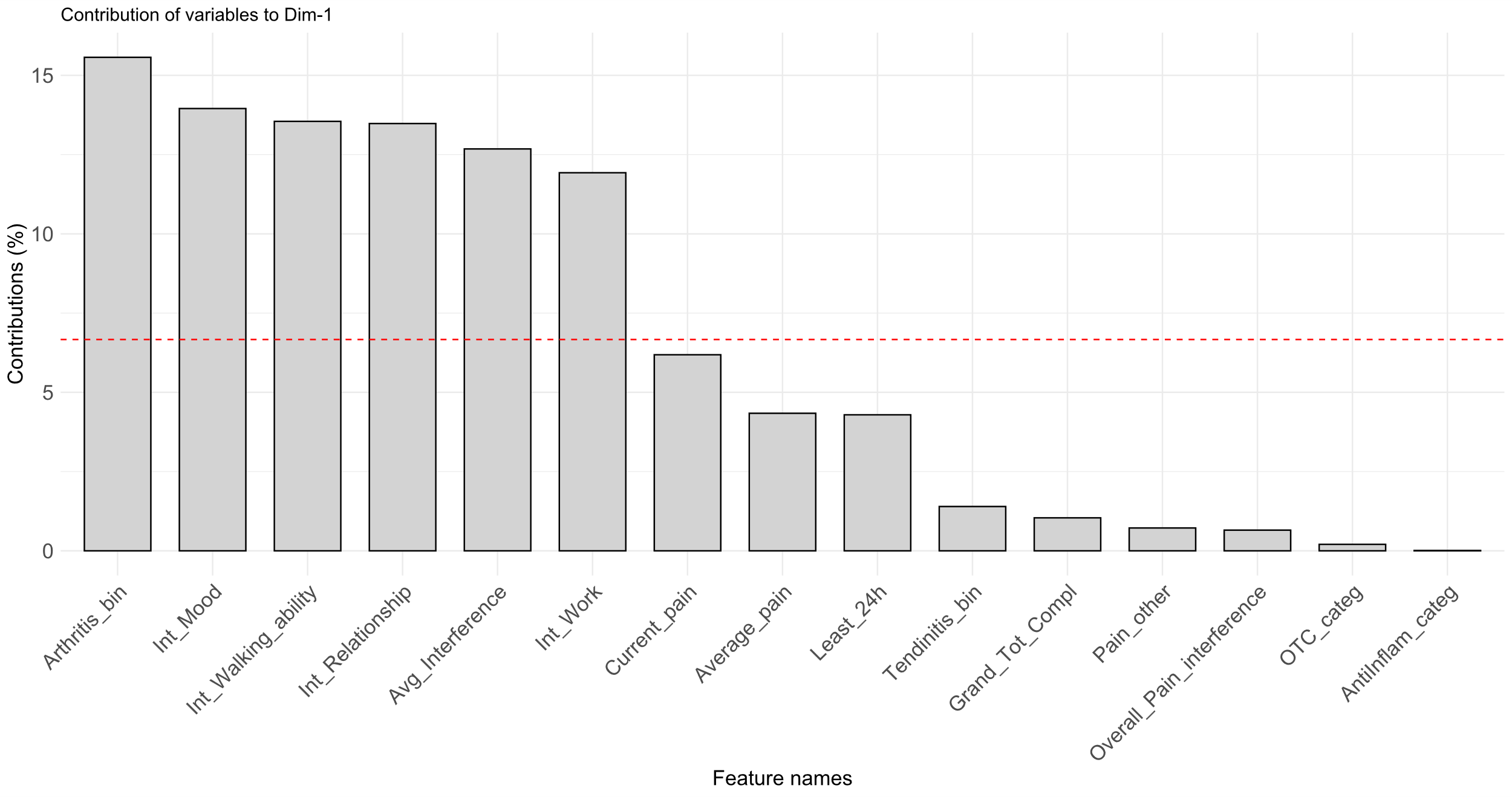


Supplementary Figure 23. Contribution of the selected by MEvA-X features in the PC1 of the OPERA dataset, Label_4 (Complaints_Change Label)


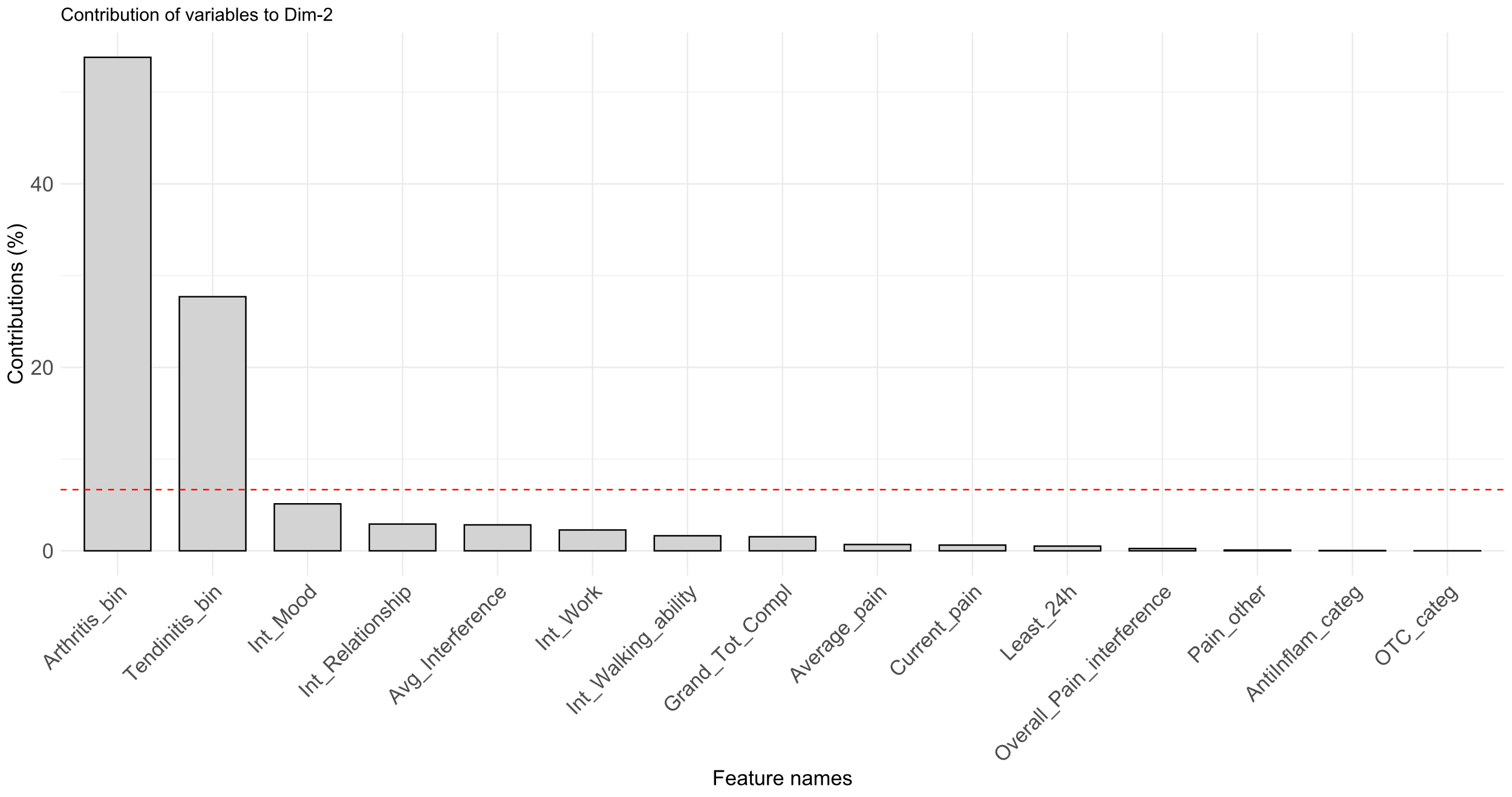


Supplementary Figure 24. Contribution of the selected by MEvA-X features in the PC2 of the OPERA dataset, Label_4 (Complaints_Change Label)


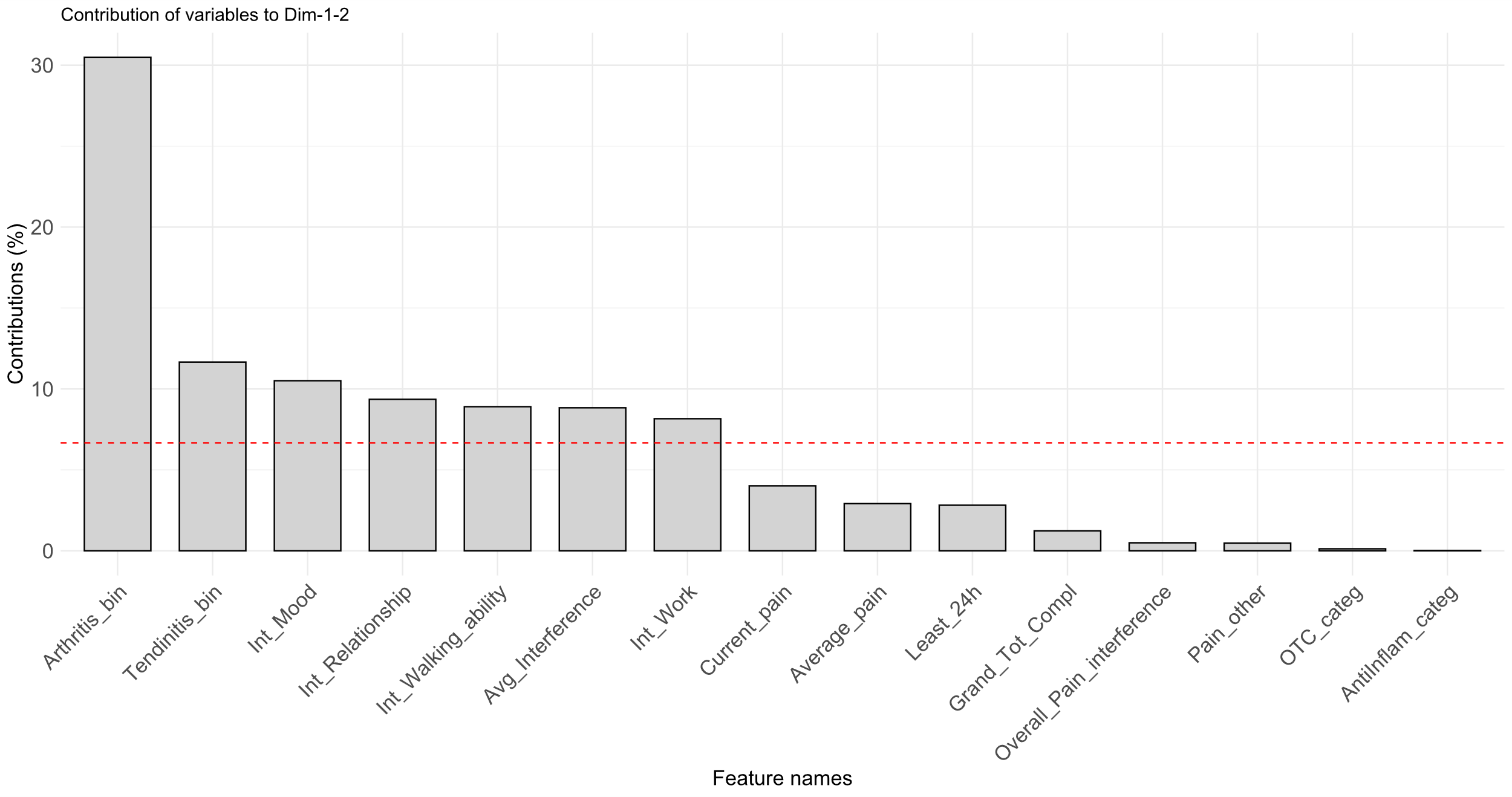


Supplementary Figure 25. Contribution of the selected by MEvA-X features in the PC1&PC2 of the OPERA dataset, Label_4 (Complaints_Change Label)

# Supplementary Tables

| **Label Name** | **Class 0** | **Class 1** | **Total** | **Ratio [0:1]** |
| --- | --- | --- | --- | --- |
| Severity Change | 506 | 125 | 631 | 4.05 |
| Interference Change | 523 | 108 | 631 | 4.84 |
| Change Grand Total Medicines | 581 | 50 | 631 | 11.62 |
| Change In Total Complaints | 481 | 150 | 631 | 3.21 |

Supplementary Table 1. Distribution of the binary classes, and the ratio between class_0 and class_1 in the four different outcomes of the OPERA study datasets.

|  | Name | Gene index | Description | Value Range (min – max) |
| --- | --- | --- | --- | --- |
| Feature Selection | FS | 0 | Feature selection method | 0 - 4 |
|  | FS type | 1 | Type of feature filtering | 0 - 3 |
|  | K-NN | 2 | K nearest neighbors in JMI & mRMR | 4 - 10 |
|  | K-Best  (SKB) | 3 | Number of features | 1 - 100 |
| XGBoost Classifier | Eta | 4 | Learning rate | 0.01 - 0.35 |
|  | Max depth | 5 | Learner maximum depth | 1 - 7 |
|  | Gamma | 6 | Pruning parameter | 0 - 10 |
|  | Lambda | 7 | Generalization (L2) parameter | 0 - 10 |
|  | Alpha | 8 | Generalization (L1) parameter | 0 - 8 |
|  | Min child weight | 9 | Pruning parameter | 0 - 15 |
|  | Scale pos weight | 10 | Imbalance correction | 0 - 5 |

Supplementary Table 2. Parameter-genes of MEvA-X. Genes [0-3] control the feature selection methods to be applied to the dataset, while the rest [4-10] are the hyper-parameters of the XGBoost algorithm.

| **Module** | **Version** |
| --- | --- |
| pandas | 1.3.4 |
| NumPy | 1.20.3 |
| mifs | 0.0.1.dev0 (<https://github.com/danielhomola/mifs>) |
| matplotlib | 3.4.3 |
| xgboost | 1.5.0 |
| scipy | 1.7.1 |
| scikit-learn | 1.1 |
| pickle | Pickle5 |

Supplementary Table 3. List of Python modules used in the development of MEvA-X and their versions.

|  | Accuracy | AUroC | Weighted GM | F2 score | Overall | Balanced Accuracy |
| --- | --- | --- | --- | --- | --- | --- |
| Baseline model  (No feature selection) | 0.62 ± 0.144 | 0.702 ± 0.153 | 0.530 ± 0.217 | 0.608 ± 0.174 | 0.583 ± 0.121 | 0.6 ± 0.147 |
| Wilcoxon | 0.607 ± 0.138 | 0.677 ± 0.165 | 0.512 ± 0.207 | 0.595 ± 0.139 | 0.571 ± 0.116 | 0.584 ± 0.143 |
| Select K best (K=100) | 0.556 ± 0.130 | 0.599 ± 0.167 | 0.422 ± 0.229 | 0.544 ± 0.131 | 0.517 ± 0.112 | 0.519 ± 0.133 |
| JMI k = 4 | 0.743 ± 0.126 | 0.877 ± 0.121 | 0.661± 0.219 | 0.732 ± 0.132 | 0.689 ± 0.109 | 0.717 ± 0.144 |
| JMI k = 5 | 0. 740 ± 0.121 | **0.880± 0.115** | 0.669 ± 0.2 | 0.730 ± 0.127 | 0.689 ± 0.104 | 0.718 ± 0.136 |
| JMI k = 6 | 0.732 ± 0.127 | 0.870 ± 0.124 | 0.669 ± 0.193 | 0.723 ± 0.131 | 0.683 ± 0.106 | 0.712 ± 0.137 |
| JMI k = 7 | 0.741 ± 0.129 | 0.871 ± 0.125 | 0.686 ± 0.193 | 0.733 ± 0.134 | 0.691 ± 0.109 | 0.725 ± 0.140 |
| JMI k = 8 | **0.749 ± 0.122** | 0.873 ± 0.120 | **0.687 ± 0.181** | **0.740 ± 0.126** | **0. 696 ± 0.102** | **0.728 ± 0.132** |
| JMI k = 9 | 0.727 ± 0.128 | 0.852 ± 0.130 | 0.67 ± 0.185 | 0.719 ± 0.131 | 0.679 ± 0.108 | 0.709 ± 0.140 |
| JMI k = 10 | 0.734 ± 0.123 | 0.85 ± 0.132 | 0.683 ± 0.168 | 0.726 ± 0.125 | 0.685 ± 0.102 | 0.718 ± 0.134 |
| mRMR k=4 | 0.73 ± 0.124 | 0.82 ± 0.130 | 0.653 ± 0.193 | 0.720 ± 0.128 | 0.674 ± 0.105 | 0.704 ± 0.136 |
| mRMR k = 5 | 0.728 ± 0.122 | 0.835 ± 0.131 | 0.675 ± 0.153 | 0.719 ± 0.125 | 0.678 ± 0.098 | 0.708 ± 0.131 |
| mRMR k = 6 | 0.74 ± 0.117 | 0.849 ± 0.123 | 0.670 ± 0.183 | 0.730 ± 0.122 | 0.685 ± 0.1 | 0.716 ± 0.131 |
| mRMR k = 7 | 0.72 ± 0.125 | 0.824 ± 0.126 | 0.646 ± 0.193 | 0.709 ± 0.130 | 0.667 ± 0.105 | 0.697 ± 0.132 |
| mRMR k = 8 | 0.741 ± 0.119 | 0.864 ± 0.123 | 0.666 ± 0.2 | 0.729 ± 0.126 | 0.687 ± 0.103 | 0.72 ± 0.134 |
| mRMR k = 9 | 0.734 ± 0.124 | 0.87 ± 0.115 | 0.653 ± 0.212 | 0.723 ± 0.131 | 0.681 ± 0.107 | 0.709 ± 0.139 |
| mRMR k = 10 | 0.733 ± 0.128 | 0.864 ± 0.119 | 0.652 ± 0.218 | 0.722 ± 0.134 | 0.68 ± 0.111 | 0.71 ± 0.146 |

Supplementary Table 4. Comparison of the XGBoost models with prior feature selection (with different parameters) and the baseline XGBoost classifier for the Ornish diet dataset. All the experiments were conducted with a stratified 10-fold Cross-validation.

|  | Accuracy | AUC | Weighted GM | F2 score | Balanced Accuracy | Feature complexity | Overall |
| --- | --- | --- | --- | --- | --- | --- | --- |
| Baseline model  (No feature selection) | 0.62 ± 0.14 | 0.7 ± 0.15 | 0.53 ± 0.22 | 0.6 ± 0.15 | 0.6 ± 0.15 | 7E-4 ± 0.00 | 0.58 ± 0.12 |
| No EA with JMI (k=5) | 0.75 ± 0.12 | **0.88 ± 0.12** | **0. 7 ± 0.16** | 0.75 ± 0.13 | 0.73 ± 0.14 | 0.17 ± 0.01 | 0. 7 ± 0.1 |
| MEvA-X Overall | **0.76 ± 0.13** | 0.76 ± 0.14 | 0.7 ± 0.19 | **0.76 ± 0.14** | **0.74 ± 0.14** | **0.76 ± 0.11** | **0.76 ± 0.15** |
| MEvA-X Majority Voting | 0.75 ± 0.13 | 0.76 ± 0.14 | 0.69 ± 0.17 | 0.74 ± 0.13 | 0.73 ± 0.14 | 0.34 ± 0.19 | 0.74 ± 0.13 |

Supplementary Table 5. Comparative results of MEvA-X with the baseline (simple XGBoost) and the best feature selection technique (JMI k=5) with XGBoost for the Ornish diet dataset

| Parameters | | | | Evaluation metrics | | | | | | |
| --- | --- | --- | --- | --- | --- | --- | --- | --- | --- | --- |
|  |  |  |  | AUC | | F1 | | Balanced Accuracy | | |
| # Estimators | Max Depth | Min sample split | Min sample leaf | mean | Std test | mean | Std test | mean | Std test | rank |
| 145 | 2 | 3 | 4 | 0.59 | 0.26 | 0.64 | 0.17 | 0.61 | 0.18 | 1 |
| 133 | 4 | 4 | 7 | 0.59 | 0.28 | 0.63 | 0.15 | 0.61 | 0.17 | 2 |
| 84 | 8 | 8 | 8 | 0.60 | 0.22 | 0.63 | 0.15 | 0.60 | 0.17 | 3 |
| 424 | 2 | 7 | 3 | 0.59 | 0.25 | 0.63 | 0.14 | 0.60 | 0.16 | 4 |
| 740 | 3 | 2 | 1 | 0.55 | 0.26 | 0.63 | 0.14 | 0.60 | 0.16 | 5 |

Supplementary Table 6. Table of the results of the trained models with the Random Forest classification algorithm on the Ornish Dataset in a stratified 10-fold cross-validation framework using grid search for the optimization of the parameters. 5 runs were performed and the best-performing solution in each run is described in each row of the table.

|  |  | Accuracy | AUC | Weighted GM | F2 score | Balanced Accuracy | Feature complexity | Overall |
| --- | --- | --- | --- | --- | --- | --- | --- | --- |
| Label 1: “Total Severity Change” | Baseline | **0.78 ± 0.03** | **0.72 ± 0.06** | 0.38 ± 0.14 | **0.76 ± 0.03** | 0.55 ± 0.05 | 0.17 ± 0.0 | 0.62 ± 0.04 |
|  | MEvA-X | 0.69 ± 0.05 | 0.7 ± 0.07 | **0.63 ± 0.08** | 0.69 ± 0.05 | **0.64 ± 0.07** | **0.65 ± 0.0** | **0.7 ± 0.04** |
| Label 2: “Interference Change” | Baseline | **0.82 ± 0.03** | **0.78 ± 0.06** | 0.44 ± 0.15 | **0.81 ± 0.04** | 0.59 ± 0.06 | 0.16 ± 0.0 | 0.67 ± 0.05 |
|  | MEvA-X | 0.77 ± 0.04 | 0.77 ± 0.05 | **0.67 ± 0.09** | 0.77 ± 0.05 | **0.69 ± 0.07** | **0.84 ± 0.0** | **0.78 ± 0.04** |
| Label 3: “Grant Total Medicine Change” | Baseline | **0.92 ± 0.02** | **0.82 ± 0.09** | 0.27 ± 0.25 | **0.9 ± 0.02** | 0.56 ± 0.07 | 0.16 ± 0.0 | 0.71 ± 0.05 |
|  | MEvA-X | 0.88 ± 0.03 | 0.78 ± 0.08 | **0.59 ± 0.15** | 0.89 ± 0.03 | **0.67 ± 0.09** | **0.79 ± 0.0** | **0.81 ± 0.04** |
| Label 4: “Total Complaints Change” | Baseline | **0.77 ± 0.04** | **0.76 ± 0.06** | 0.54 ± 0.09 | **0.76 ± 0.04** | 0.62 ± 0.06 | 0.02 ± 0.0 | 0.64 ± 0.04 |
|  | MEvA-X | 0.68 ± 0.06 | 0.75 ± 0.07 | **0.7 ± 0.06** | 0.68 ± 0.06 | **0.71 ± 0.06** | **0.67 ± 0.0** | **0.72 ± 0.04** |

Supplementary Table 7. Comparative results between MEvA-X and the baseline (simple XGBoost) for the OPERA dataset. The lines are grouped by the four different labels of the dataset.

|  | **Label 1** | **Label 2** | **Label 3** | **Label 4** |
| --- | --- | --- | --- | --- |
|  | **Severity Change** | **Interference Change** | **Total Drug Change** | **Complaints change** |
| 1 | TFFC3 | TFFC2 | NeuroRadic_cat | Arthritis_bin |
| 2 | Age | TFFC3 | Arthritis_bin | Tendinitis_bin |
| 3 | Gender | TFFC4 | Int_Relationship | Grand_Tot_Compl |
| 4 | Other_bin | Gender | Int_Life_enjoyment | Pain_other |
| 5 | Pain_other | Arthritis_categ | Narcotic_weight | Least_24h |
| 6 | Average_pain | MyoMuscul_cat | Grand_Tot_Med_weight | Average_pain |
| 7 | Current_pain | Tendinitis_bin | Narcotic_bin | Current_pain |
| 8 | Severity_Score | Grand_Tot_Compl | Tot_Compl_categ | Overall_Pain_interference |
| 9 | Int_Work | Worst_24h |  | Int_Mood |
| 10 | Int_Sleep | Average_pain |  | Int_Walking_ability |
| 11 | Life_enjoyment | Severity_Score |  | Int_Work |
| 12 | Avg_Interference | Int_Mood |  | Int_Relationship |
| 13 | Narcotic_weight | Int_Work |  | Avg_Interference |
| 14 | Grand_Tot_Med_weight | Int_Sleep |  | OTC_categ |
| 15 |  | Avg_Interference |  | AntiInflam_categ |
| 16 |  | AntiInflam_categ |  |  |
| 17 |  | Opioid_comb |  |  |
| 18 |  | Grand_Tot_Med_weight |  |  |

Supplementary Table 8. Lists of selected features from the MEvA-X algorithm for the labels of the OPERA dataset

| **The original name of the variable** | **Alias used** |
| --- | --- |
| AgeatSurvey1 | Age |
| GenderRECODE | Gender |
| Q10S1Least | Least_24h |
| Q11S1Average | Average_pain |
| Q12S1RightNow | Current_pain |
| Q13S1 | Overall_Pain_interference |
| Q14S1GeneralActivity | Int_Gen_Activity |
| Q15S1Mood | Int_Mood |
| Q16S1WalkingAbility | Int_Walking_ability |
| Q17S1NormalWork | Int_Work |
| Q18S1RelationshipsWithOtherPeople | Int_Relationship |
| Q19S1Sleep | Int_Sleep |
| Q1S1ArthritisRecode | Arthritis_bin |
| Q1S1ArthritisTotal | Arthritis_categ |
| Q20S1EnjoymentofLife | Int_Life_enjoyment |
| Q26S1 | Meds_3days |
| Q272829S1HowManyCategoriesOfMeds | Categ_of_meds |
| Q27S1OTCRecode | OTC_bin |
| Q27S1OTCTotal | OTC_categ |
| Q28S1AntiInflamREcode | AntiInflam_bin |
| Q28S1AntiInflamTotal | AntiInflam_categ |
| Q28S1AntiInflamWeight5 | AntiInflam_weight |
| Q29S11or2OpioidsNoAnticonvulsants | Opioids_No_Anticonvulsants |
| Q29S1NarcoticRecode | Narcotic_bin |
| Q29S1NarcoticTotal | Narcotic_categ |
| Q29S1NarcoticWeight10 | Narcotic_weight |
| Q2S1NeuroRadicRecode | NeuroRadic_bin |
| Q2S1NeuroRadicTotal | NeuroRadic_cat |
| Q3S1MyoMusculPainORSpasmTotal | MyoMuscul_cat |
| Q3S1MyoMuscuRecode | MyoMuscul_bin |
| Q4S1TendinitisRecode | Tendinitis_bin |
| Q4S1TendinitisTotal | Tendinitis_cat |
| Q5S1OtherRecode | Other_bin |
| Q5S1OtherTotal | Other_cat |
| Q7S1 | Pain_other |
| Q9S1Worst | Worst_24h |
| S1GrandTotalMedicines | Grand_Tot_Med |
| S1GrandTotalMedicinesWEIGHTED | Grand_Tot_Med_weight |
| S1GrandTotalofAllComplaintsExceptOverallOther | Grand_Tot_Compl |
| S1InferenceScoreOutof10 | Avg_Interference |
| S1NotOnAntiConvulsant | Not_on_Anticonvulsant |
| S1NumberofPainCategoriesIncludingOther | Pain_categ |
| S1OnOpioidAloneorOpioidPlus | Opioid_comb |
| S1Q29OpioidAloneorOthers | Opioid_others |
| S1SeverityScoreOutof10 | Severity_Score |
| S1TotalComplaintCategoriesNotOther | Tot_Compl_categ |
| TFourFormulationCategories_1 | TFFC1 |
| TFourFormulationCategories_2 | TFFC2 |
| TFourFormulationCategories_3 | TFFC3 |
| TFourFormulationCategories_4 | TFFC4 |

Supplementary Table 9. Alias being used for the OPERA dataset to make it easier for interpretation.

# References

1. Pedregosa, F. *et al.* Scikit-learn: Machine Learning in Python. *Journal of Machine Learning Research* **12**, 2825–2830 (2011).

2. Keerin, P., Kurutach, W. & Boongoen, T. Cluster-based KNN missing value imputation for DNA microarray data. in *2012 IEEE International Conference on Systems, Man, and Cybernetics (SMC)* 445–450 (2012). doi:10.1109/ICSMC.2012.6377764.

3. Bisong, E. *Building machine learning and deep learning models on Google cloud platform: A comprehensive guide for beginners*. (Apress, 2019).

4. Al-Salami, N. M. A. Evolutionary algorithm definition. *American J. of Engineering and Applied Sciences* **2**, 789–795 (2009).

5. Reddy, M. J. & Kumar, D. N. Optimal Reservoir Operation Using Multi-Objective Evolutionary Algorithm. *Water Resources Management* **20**, 861–878 (2006).

6. Blackburn, H. L. *et al.* Gene expression profiling during intensive cardiovascular lifestyle modification: Relationships with vascular function and weight loss. *Genom Data* **4**, 50–53 (2015).

7. Ellsworth, D. L. *et al.* Intensive Cardiovascular Risk Reduction Induces Sustainable Changes in Expression of Genes and Pathways Important to Vascular Function. *Circ Cardiovasc Genet* **7**, 151–160 (2014).

8. Gudin, J. A. *et al.* Changes in pain and concurrent pain medication use following compounded topical analgesic treatment for chronic pain: 3- and 6-month follow-up results from the prospective, observational Optimizing Patient Experience and Response to Topical Analgesics study. *J Pain Res* **10**, 2341–2354 (2017).
